# Gefitinib (an EGFR tyrosine kinase inhibitor) plus anlotinib (an multikinase inhibitor) for untreated, *EGFR-*mutated, advanced non-small cell lung cancer (FL-ALTER): a multicenter phase III trial

**DOI:** 10.1038/s41392-024-01927-9

**Published:** 2024-08-13

**Authors:** Hua-Qiang Zhou, Ya-Xiong Zhang, Gang Chen, Qi-Tao Yu, Hua Zhang, Guo-Wu Wu, Di Wu, Ying-Cheng Lin, Jun-Fei Zhu, Jian-Hua Chen, Xiao-Hua Hu, Bin Lan, Ze-Qiang Zhou, Hai-Feng Lin, Zi-Bing Wang, Xiao-Lin Lei, Suo-Ming Pan, Li-Ming Chen, Jian Zhang, Tian-Dong Kong, Ji-Cheng Yao, Xin Zheng, Feng Li, Li Zhang, Wen-Feng Fang

**Affiliations:** 1 Department of Medical Oncology, Sun Yat-sen University Cancer Center, State Key Laboratory of Oncology in South China, Guangdong Provincial Clinical Research Center for Cancer, Collaborative Innovation Center for Cancer Medicine, Guangzhou, 510060 China; 2 Department of Medical Oncology of Respirotary, Affiliated Tumor Hospital of Guangxi Medical University, Nanning, 530021 China; 3 Department of Urogenital Oncology, the First People’s Hospital of Foshan, Foshan, 52800 China; 4 Department of Medical Oncology, Meizhou People’s Hospital (Huangtang Hospital), Meizhou, 514031 China; 5 Department of Respiratory and Critical Care Medicine, Shenzhen people’s Hospital, Shenzhen, 518020 China; 6 Department of Medical Oncology of Respirotary, Cancer Hospital of Shantou University Medical College, Shantou, 515031 China; 7 Department of Respiratory and Critical Care Medicine, Taizhou Central Hospital, Taizhou, 318000 China; 8 Department of Medical Oncology, Hunan Provincial Cancer Hospital, Changsha, 410031 China; 9 Department of Medical Oncology, the First Affiliated Hospital of Guangxi Medical University, Nanning, 530021 China; 10 Department of Cardiothoracic Surgery, Shantou Central Hospital, Shantou, 515031 China; 11 Department of Oncology, the 2nd People’s Hospital of Shenzhen, Shenzhen, 518025 China; 12 Department of Medical Oncology, the Second Affiliated Hospital of Hainan Medical University, Haikou, 570216 China; 13 Department of Immunotherapy, Henan Cancer Hospital, Zhengzhou, 450003 China; 14 Department of Oncology, Affiliated Hospital of Panzhihua University, Panzhihua, 617099 China; 15 Department of Radiotherapy, Yuebei People’s Hospital, Shaoguan, 512099 China; 16 Department of Oncology, the First Affiliated Hospital of Shantou University Medicine College, Shantou, 515041 China; 17 Department of Oncology, ZhuJiang Hospital of Southern Medical University (The Second Clinical Medical College), Guangzhou, 510280 China; 18 Department of Respiratory Oncology, the Third People’s Hospital of Zhengzhou, Zhengzhou, 450001 China; 19 Shanghai OrigiMed Co., Ltd, Shanghai, China

**Keywords:** Oncology, Cancer

## Abstract

Dual inhibition of vascular endothelial growth factor and epidermal growth factor receptor (EGFR) signaling pathways offers the prospect of improving the effectiveness of EFGR-targeted therapy. In this phase 3 study (ClinicalTrial.gov: NCT04028778), 315 patients with treatment-naïve, *EGFR-*mutated, advanced non-small cell lung cancer (NSCLC) were randomized (1:1) to receive anlotinib or placebo plus gefitinib once daily on days 1–14 per a 3-week cycle. At the prespecified final analysis of progression-free survival (PFS), a significant improvement in PFS was observed for the anlotinib arm over the placebo arm (hazards ratio [HR] = 0.64, 95% CI, 0.48–0.80, *P* = 0.003). Particularly, patients with brain metastasis and those harboring *EGFR* amplification or high tumor mutation load gained significant more benefits in PFS from gefitinib plus anlotinib. The incidence of grade 3 or higher treatment-emergent adverse events was 49.7% of the patients receiving gefitinib plus anlotinib *versus* 31.0% of the patients receiving gefitinib plus placebo. Anlotinib plus gefitinib significantly improves PFS in patients with treatment-naïve, *EGFR-*mutated, advanced NSCLC, with a manageable safety profile.

## Introduction

Lung cancer remains a principal cause of cancer death in both sexes globally.^1^ China is experiencing an increasing burden of lung cancer, with approximately 820,000 new cases and 720,000 deaths in 2020, accounting for 40% of global lung cancer death.^2^ Approximately 60% of lung cancer cases harbor driver alterations and targeting actionable oncogenic driver alterations remains a cornerstone in targeted therapy for non-small cell lung cancer (NSCLC),^3^ which accounts for the majority of lung cancer cases.

The epidermal growth factor receptor (*EGFR*) gene is the most frequent genetic driver in metastatic NSCLC,^4^ and, therefore, a rational therapeutic target. Several advanced clinical trials have demonstrated that EGFR tyrosine kinase inhibitors (TKIs), including gefitinib,^5,6^ the first or second EGFR TKIs, and osimertinib, a third generation EGFR-TKI,^7^ confer progression-free survival (PFS) benefit in patients with NSCLC harboring *EGFR* mutation. EGFR-TKIs have been established as standard first-line treatments for *EGFR*-mutated NSCLC.^8^ However, acquired resistance to EGFR-TKIs inevitably develops and NSCLC patients ultimately experience disease progression,^9–11^ highlighting the importance of exploring novel EGFR-TKIs or therapeutic agents exhibiting biological synergy with EGFR-TKIs for advanced *EGFR* mutated NSCLC.^12^

The vascular endothelial growth factor (VEGF) pathway plays a critical role in driving oncoangiogenesis in lung cancer and dual inhibition of VEGF signaling and EGFR signaling pathways offers the prospect of improving the effectiveness of EFGR-targeted therapy and overcoming EGFR-TKI resistance.^13^

Anlotinib is an oral multikinase inhibitor and suppresses oncoangiogenesis and tumor growth *via* blocking VEGFR, fibroblast growth factor receptor (FGFR), platelet-derived growth factor receptor (PDGFR), and c-Kit^14^ while bevacizumab only targets the VEGFR signaling pathway.^15^ The antitumor activities of anlotinib is supported by its remarkable anti-angiogenic activities compared with other TKIs, with a low IC_50_ for VEGFR-2 (0.2 nmol/L *vs*. lenvatinib 4 nmol/L and sorafenib 90 nmol/L) and VEGFR-3 (0.7 nmol/L *vs*. lenvatinib 5.2 nmol/L and sorafenib 20 nmol/L).^15–19^ Currently, it is the only approved anti-angiogenic drug for lung cancer in China and has been recommended as third and later-line treatment for advanced NSCLC based on the ALTER 0303 trial showing anlotinib significantly extending the overall survival (OS) (HR 0.68, 95% CI 0.54–0.87) and PFS (HR 0.25, 95% CI 0.19–0.31) of advanced NSCLC patients progressing upon second or further line treatment.^20,21^ A subgroup analysis of the ALTER 0303 trial showed that anlotinib led to a 79% reduction in the risk of progression (hazard ratio [HR] 0.21; 95% confidence interval [CI]: 0.13–0.32) a 41% reduction in the risk of death (HR 0.59; 95% CI: 0.38–0.94) versus placebo in treatment-naïve NSCLC patients and this benefit was independent of *EGFR* mutation status.^22^ Anlotinib also showed promising antitumor activities in the first-line setting for advanced NSCLC in combination with another EGFR-TKI^23^ and immune checkpoint inhibitor sintilimab.^24^

Anlotinib offers convenient oral dosing compared to intravenous infusion for currently available anti-angiogenic inhibitors and has been studied in the first-line setting for NSCLC plus chemotherapy.^25^ We hypothesized that anlotinib in combination with an EGFR-TKI would be more effective than EGFR-TKI monotherapy for advanced NSCLC in the first-line setting. In this study, we sought to investigate the efficacy and safety of gefitinib plus anlotinib for previously untreated Chinese patients with *EGFR*-mutated advanced NSCLC.

## Results

### Patient characteristics

**Fig. 1:**
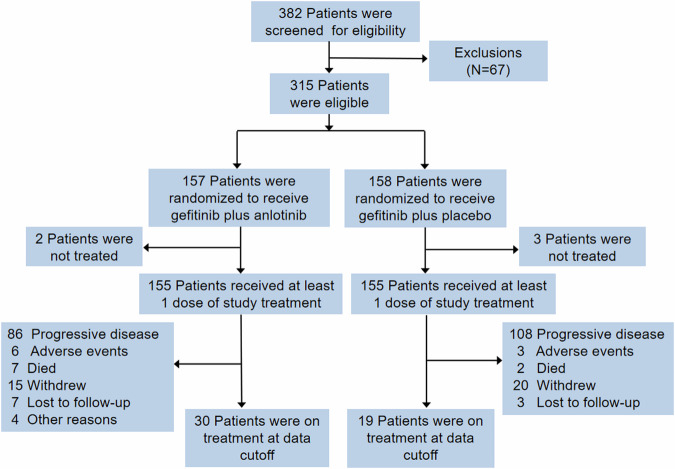
The study flowchart and patient disposition. Enhancement MRI was performed during screening to evaluate each patient for brain metastasis and MRI/CT scans were undertaken in patients with brain metastasis at baseline during follow up and in other patients at the discretion of the investigators

Between April 2019 and August 2021, 382 patients were screened for eligibility and 315 patients were eligible for the study and underwent randomization to receive gefitinib plus anlotinib (*n* = 157) or gefitinib plus placebo (*n* = 158) (Fig. 1). One hundred fifty-five patients in each group received at least one dose of the study medication and were included in the FAS. At the data cutoff (July 31, 2022), treatment was still ongoing for 30 (19.1%) patients in the gefitinib plus anlotinib group and 19 (12.0%) in the gefitinib plus placebo group.

The patients had a median age of 59 years and 43.9% were male. Most patients (92.9%) had clinical stage IV disease. Thirty-four (11.0%) patients had an ECOG performance status of 0 and 263 (84.8%) had an ECOG performance status of 1. Ninety-nine (31.9%) patients had brain metastasis and 42 (13.5%) had liver metastasis; at least 2 organs were involved in 196 (63.2%) patients. One hundred sixty-one (51.9%) patients harbored *EGFR* ex19del mutation and 149 (48.1%) harbored *EGFR* ex21L858R mutation (Table 1).

**Table 1:** Patient demographic and baseline characteristics-full analysis set

Characteristics	Gefitinib plus anlotinib (*n* = 155)	Gefitinib plus placebo (*n* = 155)
Age, years		
Median (range)	59.0 (29.0–77.0)	59.0 (32.0–76.0)
<65	119 (76.8)	108 (69.7)
≥65	36 (23.2)	47 (30.3)
Male sex	68 (43.9)	67 (43.2)
ECOG performance status score		
0	19 (12.3)	15 (9.7)
1	130 (83.9)	133 (85.8)
Unknown	6 (3.4)	7 (4.5)
Smoking status		
Never smokers	118 (76.1)	121 (78.1)
Former smokers	26 (16.8)	24 (15.5)
Current smokers	10 (6.5)	9 (5.8)
Unknown	1 (0.7)	1 (0.7)
Stage of disease		
IIIB	10 (6.5)	4 (2.6)
IV	142 (91.6)	148 (95.5)
Unknown	3 (1.9)	3 (1.9)
Tumor histologic subtypes		
Adenocarcinoma	153 (98.7)	150 (96.8)
Others	2 (1.3)	5 (3.2)
Type of *EGFR* mutations		
Exon 19 deletion	80 (51.6)	81 (52.3)
L858R	75 (48.4)	74 (47.7)
Metastatic sites		
≤2	54 (34.8)	58 (37.4)
>2	101 (65.2)	95 (61.3)
Unknown	0 (0.0)	2 (1.3)
Brain metastasis		
Yes	49 (31.6)	50 (32.3)
No	106 (68.3)	105 (67.7)
Liver metastasis		
Yes	20 (12.9)	22 (14.2)
No	135 (87.1)	133 (85.8)

Data are expressed as number (%) unless otherwise indicated

Eastern Cooperative Oncology Group (ECOG) performance-status (PS) scores range from 0 to 5, with higher numbers indicating increasing impairment in activities of daily living

Definitions: Never smokers, defined as smoking <100 cigarettes/lifetime; former smokers, defined as abstinence from smoking for at least 15 years on the day before the start of therapy; current smokers, defined as smoking >100 cigarettes/lifetime, or smoking >100 cigarettes/lifetime but abstinence from smoking for less than one year on the day before the start of therapy

### Efficacy analysis

**Fig. 2:**
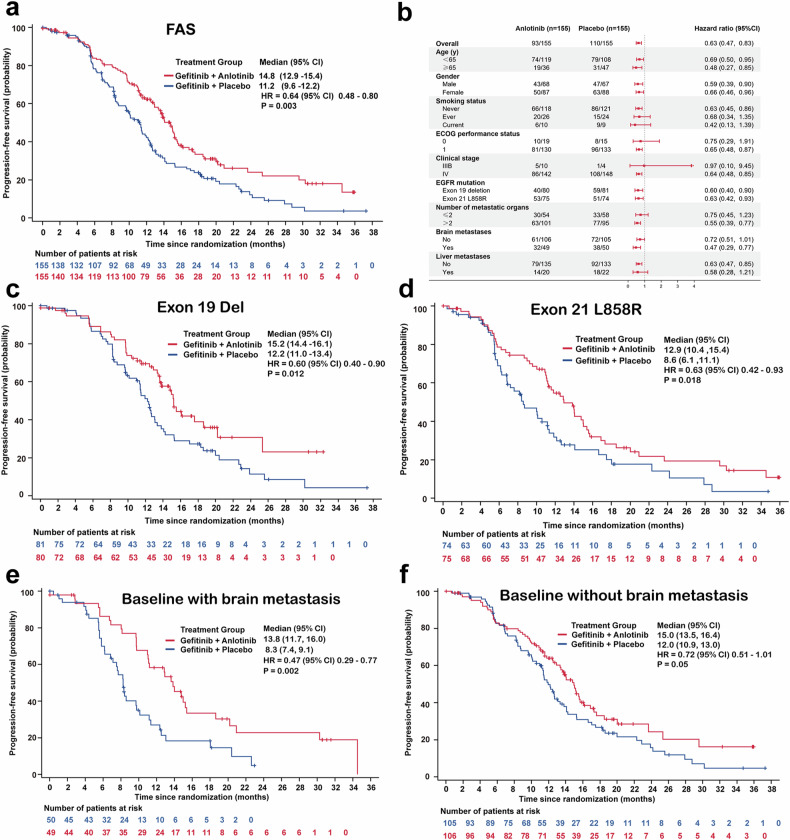
Gefitinib plus anlotinib improves progression-free survival (PFS) of advanced NSCLC patients. **a** The Kaplan–Meier curves PFS of advanced NSCLC patients treated with gefitinib plus anlotinib or gefitinib plus placebo in the FAS. **b** Forest plots for PFS. The Kaplan–Meier curves of PFS of patients harboring *EGFR* Exon 19 Del **c** or *EGFR* Exon 21 L858R **d** treated with gefitinib plus anlotinib or gefitinib plus placebo. **e** The Kaplan–Meier curves of PFS of patients with **e** or without **f** brain metastasis treated with gefitinib plus anlotinib or gefitinib plus placebo

Patients were followed up for median duration of 18.5 (95% CI 15.9–20.1) months in the gefitinib plus anlotinib group and 18.2 months (95% CI 15.5–19.9) in the gefitinib plus placebo group. As of the data cutoff date, 99 and 114 independent review committee (IRC)-confirmed PFS events had occurred in the gefitinib plus anlotinib group and the gefitinib plus placebo group, respectively. The median PFS was 14.8 months (95% CI, 12.9–15.4) in the gefitinib plus anlotinib *group versus* 11.2 months (95% CI, 9.6–12.2) in the gefitinib plus placebo group (HR = 0.64, 95% CI 0.48–0.80; stratified log rank test, *P* = 0.003) (Fig. 2a and Supplementary table 1). Diverse groups of NSCLC patients gained significant benefit in PFS from gefitinib plus anlotinib (Fig. 2b). In patients with *EGFR* ex19del, gefitinib plus anlotinib conferred significantly greater benefit in PFS than gefitinib plus placebo (15.2 months, 95% CI, 14.4–16.1 vs. 12.2 months, 95% CI, 11.0–13.4; HR = 0.60, 95% CI, 0.40–0.90) (Fig. 2c). Furthermore, patients harboring *EGFR* ex21L858R gained significant PFS benefit from gefitinib plus anlotinib than gefitinib plus placebo (12.9 *vs*. 8.6 months, HR = 0.63, 95% CI, 0.42–0.93) (Fig. 2d). Notably, among patients with brain metastasis, gefitinib plus anlotinib extended the median PFS by 5.5 months *versus* gefitinib plus placebo, with a 53% reduction in the risk of progression (13.8 *vs*. 8.3 months; HR = 0.47, 95% CI, 0.29–0.77; log rank test *P* = 0.002) (Fig. 2e). Besides, patients without brain metastasis receiving gefitinib plus anlotinib tended to have a longer PFS than those receiving gefitinib plus placebo (15.0 *vs*. 12.0 months; HR = 0.72, 95% CI, 0.51–1.01; log rank test *P* = 0.05) (Fig. 2f).

Thirty-eight deaths occurred in the gefitinib plus anlotinib group, and 33 deaths were reported in the gefitinib plus placebo group. Gefitinib plus anlotinib did not significantly improved OS *versus* gefitinib plus placebo in advanced NSCLC patients (HR = 1.12, 95% CI, 0.70–1.78). The median OS was 31.2 months (95% CI, 25.7-not estimable [NE]) in patients receiving gefitinib plus anlotinib and not reached in the gefitinib plus placebo group (95% CI, 27.1-NE; stratified log rank test, *P* = 0.644) (Supplementary Fig. 1a). No statistical difference was observed in OS across all subgroups of the two groups (Supplementary Fig. 1b).

In the FAS population, the objective response rate was significantly higher with gefitinib plus anlotinib (76.1%, 95% CI, 68.6–82.6%) *versus* gefitinib plus placebo (64.5%, 95% CI, 56.4–72.0%) (chi-square test, *P* = 0.025) (Supplementary Table 1 and Supplementary Fig. 2). The median duration of response was remarkably longer with gefitinib plus anlotinib (12.5 months, 95% CI, 11.1–16.2) *versus* that with gefitinib plus placebo (9.5 months, 95% CI 7.0–10.3) (log rank test *P* < 0.001).

### Dynamic analysis of peripheral blood ctDNA

**Fig. 3:**
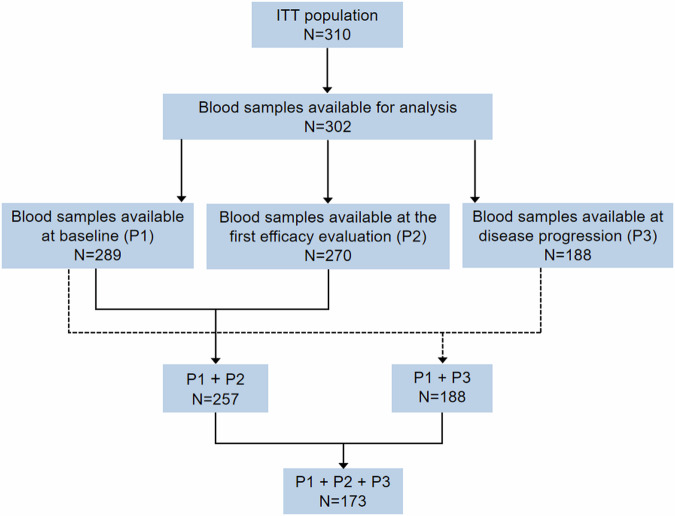
The flowchart for next generation sequencing (NGS) analysis. P1, baseline, P2, first posttreatment efficacy evaluation and P3, progressive disease

**Fig. 4:**
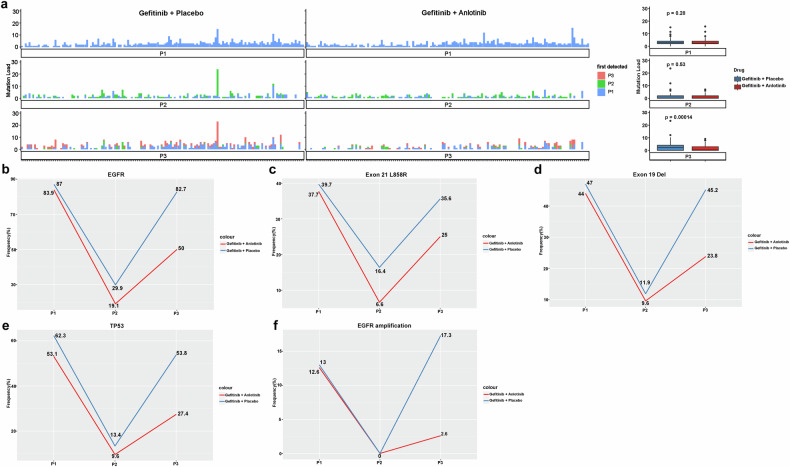
Dual inhibition of VEGF and EGFR signaling pathways reduces the tumor mutational load and the frequencies of key driver gene mutations. **a** Tumor mutational load in individual patients at baseline (P1), the first posttreatment efficacy evaluation (P2) and disease progression (P3) (left panel). Each column represents one patient in efficacy evaluable patients. Boxplots of tumor mutational load in patients in the gefitinib plus anlotinib group and the gefitinib plus placebo group are shown on the right. Temporal changes in the rates of mutated *EGFR* gene **b**, *EGFR* Exon 21 L858R **c** and Exon 19 Del **d**, mutated *TP53* gene **e** and copy number variations (amplification) **f** in the two groups from baseline to disease progression

We hypothesized that dual inhibition of VEGF and EGFR signaling pathways could be more effective in suppressing the frequencies of mutated key driver genes than TKI monotherapy. We analyzed the genomic data based on blood samples for circulating tumor DNA (ctDNA) analysis which were available for 289 patients at baseline, 270 at the first posttreatment efficacy evaluation and 188 at the time of disease progression (Fig. 3). Next generation sequencing (NGS) revealed no notable difference in the median tumor mutational load (TML) between the two groups at baseline (gefitinib plus anlotinib 3.0 *vs*. gefitinib plus placebo 3.0, *P* = 0.30, single nucleotide variation) and the first posttreatment efficacy evaluation (gefitinib plus anlotinib 1.0 *vs*. gefitinib plus placebo 1.0, *P* = 0.53). At the time of progression, patients in the gefitinib plus anlotinib group had a significantly lower TML than patients in the gefitinib plus placebo group (2.0 *vs*. 4.0, *P* < 0.001) (Fig. 4a).

*EGFR* mutations were detectable by peripheral blood ctDNA in 83.9% of the patients receiving gefitinib plus anlotinib *vs*. 87.0% of those receiving gefitinib plus placebo at baseline. At the first posttreatment efficacy evaluation, the rate of mutated *EGFR* with gefitinib plus anlotinib was lower than that with gefitinib plus placebo (19.1% *vs*. 29.9%). Meanwhile, upon disease progression, it approached the level before treatment in the gefitinib plus placebo group (82.7%) while remaining subdued in the gefitinib plus anlotinib group (50.0%) (Fig. 4b). A similar trajectory of changes was observed in *EGFR* ex21L858R and ex19del (Fig. 4c, d).

The presence of mutated *TP53* is reported to be an adverse predictor of TKI therapeutic outcome in NSCLC.^26^ We next examined the temporal changes in the mutational profiles of *TP53*, an established tumor initiator gene. The rate of mutated *TP53* also showed a similar trajectory of changes to that of mutated *EGFR*, with a notable reduction in mutated *TP53* rate in both groups at the first posttreatment efficacy evaluation (gefitinib plus anlotinib 9.6% from 53.1% at baseline *vs*. gefitinib plus placebo 13.4% from 62.3% at baseline). The rate of mutated *TP53* approached the baseline level in patients receiving gefitinib plus placebo at the time of disease progression but only showed a modest rise in patients receiving gefitinib plus anlotinib (Fig. 4e).

In addition, *EGFR* amplification occurred in 12.6% of the patients in the gefitinib plus anlotinib group at baseline and was not detected at the first efficacy evaluation and rose to 2.6% at PD. Meanwhile, *EGFR* amplification occurred in 13.0% of the patients in the gefitinib plus placebo group at baseline and were undetected at the first efficacy evaluation; upon disease progression, it increased to 17.3%. The rate of *EGFR* amplification at the time of PD was significantly higher in the gefitinib plus placebo group than the gefitinib plus anlotinib group (*P* < 0.001) (Fig. 4f).

### Biomarkers analysis

**Fig. 5:**
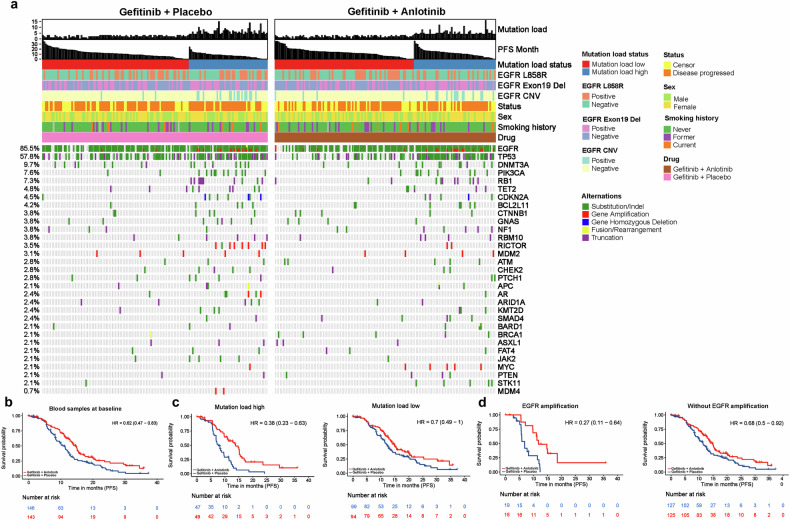
Dual inhibition of VEGF and EGFR signaling pathways derives more benefits in patients with *EGFR* amplification or a high tumor mutational load. **a** Heatmaps show frequent somatic mutations (≥2%) in pretreatment NSCLC samples. Left, gefitinib plus placebo, right, gefitinib plus anlotinib. **b** The Kaplan–Meier PFS curves of advanced NSCLC patients with blood sample available at baseline **c** The Kaplan–Meier PFS curves of patients with high (left) and low tumor mutational load (right) treated with gefitinib plus anlotinib *versus* gefitinib plus placebo. **d** The Kaplan–Meier PFS curves of patients with (left) or without *EGFR* amplification (right) who were treated with gefitinib plus anlotinib *versus* those who were treated with gefitinib plus placebo

The mutational profiles at baseline of *EGFR-*mutated, advanced NSCLC patients receiving gefitinib and anlotinib and gefitinib plus placebo are shown in Fig. 5a. Dual therapy with gefitinib plus anlotinib significantly reduced the risk of progression in patients with blood sample available at baseline compared to gefitinib plus placebo (HR = 0.62, 95% CI 0.47–0.83, *P* = 0.001) (Fig. 5b). Currently, there are no established molecular biomarkers that can reliably predict clinical response or resistance to anti-angiogenic agents. We evaluated TML as a biomarker to estimate survival benefit associated with the combination of gefitinib and anlotinib. The median TML was 3.0 at baseline in both groups and was used to define patients with high and low TML. Gefitinib plus anlotinib extended the median PFS by 6.2 months *versus* gefitinib plus placebo (13.8 months, 95% CI 10.9–15.5 *vs*. 7.6 months, 95% CI 6.8–9.8), with a 62% reduction in the risk of progression in patients with high TML at baseline (HR = 0.38, 95% CI 0.23–0.63, *P* < 0.001) (Fig. 5c, left). Anlotinib added to gefitinib led to a 30% reduction in the risk of progression in patients with low TML at baseline (HR = 0.70, 95% CI 0.49–1.00; log rank test, *P* = 0.050), with a 2.5-month extension of PFS (14.7 months, 95% CI 12.9–20.0 *vs*. 12.2 months, 95% CI 10.6–14.3) (Fig. 5c, right).

Copy number variation (CNV) could predict response to EGFR TKI therapy in patients with advanced NSCLC.^27,28^ However, the impact of *EGFR* amplification on the efficacy of dual therapy with an anti-angiogenic agent and EGFR TKI remains undefined and was explored in this study. Patients harboring *EGFR* amplification at baseline experienced a 73% reduction in the risk of progression with gefitinib plus anlotinib (HR = 0.27, 95% CI, 0.11–0.64; log rank test, *P* = 0.002), extending the median PFS by 4.9 months *vs*. gefitinib plus placebo (11.7 months, 95% CI 9.8-NE *vs*. 6.8 months, 95% CI 5.7–12) (Fig. 5d, left). Gefitinib plus anlotinib also lowered the risk of progression in patients without *EGFR* amplification at baseline (HR = 0.68, 95% CI, 0.50–0.92; log rank test, *P* = 0.012) (Fig. 5d, right and Supplementary Fig. 3).

### *EGFR* mutations and the associated gene pathways at PD

At the time of PD, *EGFR* ex21L858R, *EGFR* ex19del and secondary *EGFR*^*T790M*^ mutation accounted for 32.8%, 31.3% and 28.1% of *EGFR* mutations in patients receiving gefitinib plus anlotinib and 22.6%, 28.7%, and 31.1% of *EGFR* mutations in patients receiving gefitinib plus placebo, respectively (Supplementary Fig. 4a). Meanwhile, *EGFR* amplification accounted for 3.1% and 11.0% of *EGFR* mutations in patients receiving gefitinib plus anlotinib and those receiving gefitinib plus placebo, respectively. In addition, the rates of mutated *EGFR*, *TP53*, and *RICTOR* were significantly lower in patients receiving gefitinib plus anlotinib than those on gefitinib plus placebo (Supplementary Fig. 4b).

**Fig. 6:**
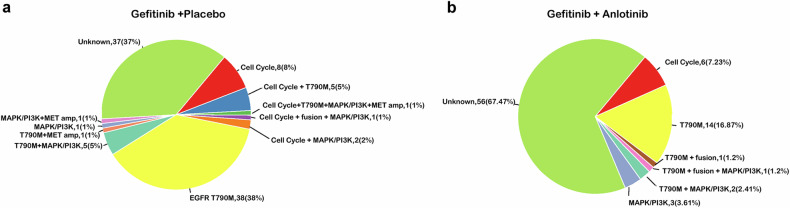
Possible resistance mechanisms at the time of disease progression. **a** The gefitinib plus placebo group; **b** The gefitinib plus anlotinib group

Gene pathway analysis showed that the mutated genes at the time of disease progression were involved in multiple cellular signaling pathways. The gefitinib plus placebo group had significantly higher rates of gene mutations implicated in EGFR signaling, p53 signaling, cell cycle progression, checkpoint factor (CPF) and RICTOR signaling than the gefitinib plus anlotinib group (Supplementary Fig. 4c). Secondary *EGFR*^*T790M*^ mutations were the predominant change in both groups, occurring in 16.9% of patients receiving gefitinib plus anlotinib *vs*. 38.0% of patients receiving gefitinib plus placebo. This was followed by aberrant cell cycle signaling (gefitinib plus anlotinib 8.0% *vs*. gefitinib plus placebo 7.2%) (Fig. 6). However, resistance mechanisms remained undefined in 67.5% of the patients in the gefitinib plus anlotinib group and 37.0% of the patients in the gefitinib plus placebo group. At the data cutoff date, among 107 patients with treatment records after disease progression, 68% (30/44) went on to receive a third generation TKI in the anlotinib arm and 78% (49/63) in the placebo arm.

### Safety

The safety set included 155 patients in the gefitinib plus anlotinib group and 155 patients in the gefitinib plus placebo group. Treatment was interrupted in 50 (32.3%) patients receiving gefitinib plus anlotinib and 34 (21.9%) patients receiving gefitinib plus placebo.

Treatment emergent AEs (TEAEs) led to dose reductions in 48 (31.0%) patients receiving gefitinib plus anlotinib and 21 (13.6%) patients receiving gefitinib plus placebo and treatment termination in 16 (10.3%) patients receiving gefitinib plus anlotinib and 7 (4.5%) patients receiving gefitinib plus placebo. Two (1.3%) patients receiving gefitinib plus anlotinib died, due to cerebral infarction and progressive tumor. One (0.7%) patient receiving gefitinib plus placebo died due to compression of the pulmonary artery. Subsequent antitumor therapies are provided in Supplementary Table 2.

Any grade TEAEs occurred in 99.4% of the patients receiving gefitinib plus anlotinib and 97.4% of the patients receiving gefitinib plus placebo. Grade 3 or higher TEAEs were reported in 49.7% of the patients receiving gefitinib plus anlotinib and 31.0% of those receiving gefitinib plus placebo. The most frequent any-grade TEAEs are shown in Table 2. The 3 most frequent any-grade TEAEs were diarrhea (66.5%), rash (65.8%) and hypertension (65.2%) in the gefitinib plus anlotinib group, and rash (52.9%), elevated alanine aminotransferase (ALT, 48.4%) and aspartate aminotransferase (AST, 48.4%) in the gefitinib plus placebo group. The most frequent grade 3 or higher TEAEs in the gefitinib plus anlotinib arm were hypertension (29.7%) and elevated ALT (6.5%). Meanwhile, the most frequent grade 3 or higher TRAEs in the gefitinib plus placebo arm were elevated ALT (12.3%) and AST (7.1%).

**Table 2:** Treatment-emergent adverse events reported in at least 10% of the patients in the two treatment arms

	Gefitinib + anlotinib (N = 155)		Gefitinib + placebo (N = 155)	
	Any grade	Grade 3 or higher	Any grade	Grade 3 or higher
Any TEAEs	154 (99.4)	-	151 (97.42)	-
Grade ≥ 3 TEAEs	-	77 (50.0)	-	48 (31.0)
Serious TEAEs	17 (11.0)	-	9 (5.8)	-
TEAEs leading to dose interruption, any drug	50 (32.3)	-	34(21.9)	-
TEAEs leading to dose reduction, any drug	48 (31.0)	-	21 (13.6)	-
Discontinued treatment due to TEAEs	16 (10.3)	-	7 (4.5)	-
$ TEAEs leading to death	2 (1.3)	-	1 (0.7)	-
TEAEs (≥ 10%)				
Diarrhea	103 (66.5)	0 (0.0)	63 (40.7)	2 (1.3)
Rash	102 (65.8)	5 (3.23)	82 (52.90)	0 (0.0)
Hypertension	101 (65.2)	46 (29.68)	42 (27.10)	8 (5.2)
ALT increased	73 (47.1)	10 (6.45)	75 (48.39)	19 (12.3)
AST increased	70 (45.2)	5 (3.23)	75 (48.39)	11 (7.1)
Proteinuria	68 (43.9)	3 (1.94)	38 (24.52)	0 (0.0)
Hypertriglyceridemia	65 (41.9)	4 (2.58)	54 (34.84)	2 (1.3)
Occult blood positive	58 (37.4)	0 (0.00)	58 (37.42)	0 (0.0)
Palmoplantar redness syndrome	51 (32.9)	3 (1.94)	24 (15.48)	0 (0.0)
Hypercholesterolemia	43 (27.7)	0 (0.00)	28 (18.06)	0 (0.0)
Dysphonia	42 (27.1)	0 (0.00)	1 (0.65)	0 (0.0)
Urine occult blood positive	41 (26.5)	0 (0.00)	35 (22.58)	2 (1.29)
Cough	35 (22.6)	0 (0.00)	34 (21.94)	0 (0.00)
Anorexia	35 (22.6)	0 (0.00)	16 (10.32)	1 (0.65)
Dizzy	35 (22.6)	0 (0.00)	25 (16.13)	1 (0.65)
Urine white blood cell positive	31 (20.0)	1 (0.65)	23 (14.84)	1 (0.65)
Mouth ulcer	29 (18.7)	0 (0.00)	17 (10.97)	0 (0.00)
Gingival bleeding	29 (18.7)	0 (0.00)	14 (9.03)	0 (0.00)
Weakness	28 (18.1)	0 (0.00)	14 (9.03)	0 (0.00)
Nosebleed	26 (16.8)	1 (0.65)	17 (10.97)	0 (0.00)
Headache	26 (16.8)	0 (0.00)	14 (9.03)	0 (0.00)
Hypokalemia	25 (16.1)	2 (1.29)	12 (7.74)	1 (0.65)
Oral mucositis	25 (16.1)	3 (1.94)	8 (5.16)	0 (0.00)
Hematuria	25 (16.1)	1 (0.65)	15 (9.68)	0 (0.00)
Vomiting	24 (15.5)	1 (0.65)	15 (9.68)	0 (0.00)
Hyperuricemia	23 (14.8)	0 (0.00)	27 (17.42)	0 (0.00)
Pruritus	23 (14.8)	0 (0.00)	27 (17.42)	0 (0.00)
Urine red blood cell positive	22 (14.2)	0 (0.00)	15 (9.68)	0 (0.00)
Back pain	21 (13.6)	0 (0.00)	27 (17.42)	0 (0.00)
Paronychia	21 (13.6)	1 (0.65)	20 (12.90)	0 (0.00)
Joint pain	20 (12.9)	0 (0.00)	14 (9.03)	0 (0.00)
Chest pain	18 (11.6)	0 (0.00)	23 (14.84)	0 (0.00)
Nasopharyngitis	17 (11.0)	0 (0.00)	17 (10.97)	0 (0.00)
Constipation	17 (11.0)	0 (0.00)	15 (9.68)	0 (0.00)
Hemoptysis	17 (11.0)	2 (1.29)	14 (9.03)	0 (0.00)
Fever	16 (10.3)	0 (0.00)	6 (3.87)	0 (0.00)
Abdominal pain	16 (10.3)	0 (0.00)	8 (5.16)	0 (0.00)
Blood bilirubin increased	16 (10.3)	0 (0.00)	22 (14.19)	1 (0.65)
Gingival pain	16 (10.3)	0 (0.00)	5 (3.23)	0 (0.00)
Limb pain	16 (10.3)	0 (0.00)	13 (8.39)	1 (0.65)

ALT, alanine aminotransferase; AST, aspartate aminotransferase; TEAEs, treatment emergent adverse events

Treatment-emergent adverse events were evaluated throughout the treatment period and to 30 days post the final dose using NCI CTC AE version 4.0 and coded using MedDRA 24.0

Gefitinib + anlotinib: 1 patient reported multiple cerebral infarctions leading to death, and 1 patient reported dyspnea leading to death

Gefitinib + placebo: 1 patient reported Pulmonary hypertension leading to death

## Discussion

This multicenter double-blind phase III trial demonstrated that addition of anlotinib to gefitinib significantly reduced the risk of progression in treatment-naïve *EGFR-*mutated, advanced NSCLC patients, conferring PFS benefits among multiple subgroups of NSCLC patients, especially those with brain metastasis and broadly active against NSCLC of diverse genomic profiles. The combination therapy showed an acceptable safety profile. The findings support continued development of gefitinib plus anlotinib in the frontline setting for advanced NSCLC patients with *EGFR* activating mutations.

In the current trial, more than half of the population were women, the majority were never smokers, most had lung adenocarcinoma and approximately one third of them had brain metastasis upon presentation. The study demonstrated that additional anti-angiogenic therapy with anlotinib, which offers convenient oral dosing instead of intravenous infusion as with bevacizumab, provides a clear benefit in terms of PFS *versus* gefitinib monotherapy for patients with advanced NSCLC harboring *EGFR* activating mutations. In the study, gefitinib plus anlotinib extended PFS by 3.6 months *versus* gefitinib plus placebo, with a 36% reduction in the risk of progression (HR = 0.64, 95% CI 0.48–0.80). The PFS gain is consistent with the Okayama Lung Cancer Study Group Trial 1001 in Japanese patients with previously untreated *EGFR*-mutated advanced NSCLC,^29^ showing that bevacizumab added to gefitinib extended PFS by 4–5 months over historical controls receiving gefitinib monotherapy.^5,30^ This benefit in PFS is also similar to the ARTEMIS-CTONG1509 study^31^ showing a significant reduction in the risk of progression with bevacizumab added to erlotinib *versus* erlotinib alone (HR = 0.55; 95% CI, 0.41–0.73). One strength of the current study is a double-blind design while the ARTEMIS-CTONG1509 study^31^ and many other trials have an open-label design in which investigators might have bias in deciding treatment continuation.^32^ Of note, a recent phase 2 study failed to exhibit the superiority of osimertinib, a third-generation irreversible EGFR TKI, plus bevacizumab over osimertinib monotherapy in improving the PFS as a front-line treatment for patients with NSCLC harboring *EGFR* mutations.^32^ Osimertinib has demonstrated superior efficacy as first-line therapy compared with first-generation TKIs erlotinib and gefitinib for *EGFR*-mutated, advanced NSCLC patients in the FLAURA trial and in combination with chemotherapy *versus* osimertinib monotherapy in the FLAURA2 trial.^7^ It also significantly extended the PFS of patients of previously untreated, *EGFR* mutation-positive advanced NSCLC compared to gefitinib (18.9 months *vs*. 10.2 months; HR for disease progression or death, 0.46; 95% CI, 0.37–0.57).^7^ However, resistance to first line osimertinib eventually emerges and the resistance mechanism are mostly non-targetable. Apart from inconvenient intravenous administrations every 3 weeks, the PFS benefit of osimertinib plus chemotherapy comes at increased toxicities. Combination strategies including amivantamab plus lazertinib in the MARIPOSA study^33^ that could overcome emerging resistance in the first-line setting for NSCLC with sensitive EGFR mutations remain to be defined.

The PFS benefit in our study did not translate into any gain in the OS of the patients across all subgroups. This is consistent with the ARTEMIS-CTONG1509 study^31^ and the NEJ026 trial.^34^ The current trial was powered for PFS as the primary endpoint, which relies on radiological assessment of disease progression and OS was still immature at the data cutoff. In addition, OS may be heavily influenced by subsequent 2^nd^ or later line treatment after disease progression.^35,36^

Our subgroup analysis demonstrated that gefitinib plus anlotinib provided significant benefits in PFS for patients with *EGFR* ex19del and *EGFR* ex21L858R *versus* gefitinib plus placebo. *EGFR* ex21L858R is a known adverse predictor of PFS.^29^ Gefitinib plus anlotinib extended the PFS of patients harboring *EGFR* ex21L858R by 4.3 months (HR = 0.63, 95% CI 0.42–0.93). Twenty to 30% of NSCLC patients have brain metastases at the time of initial diagnosis.^37^ The NEJ026 study allowed inclusion of patients with brain metastasis but demonstrated no PFS benefit with erlotinib plus bevacizumab *versus* erlotinib alone for this subgroup^38^ (HR = 0.78, 95% CI 0.42–1.43). A *post hoc* analysis of the phase 3 ALTER0303 trial showed that anlotinib as a second or later line treatment significantly reduced the risk of progression in advanced NSCLC patients with brain metastasis *versus* placebo (HR = 0.29, 95% CI 0.15–0.56), suggesting intracranial antitumor activities of anlotinib.^39^ In this trial, gefitinib plus anlotinib led to a 53% reduction in the risk of progression or death *versus* gefitinib plus placebo in patients with brain metastasis (HR = 0.47, 95% CI, 0.29–0.77), which is consistent with the ARTEMIS-CTONG1509 study.^31^ Similar to the ALTER0303 trial and the ARTEMIS-CTONG1509 study, a recent phase 2 trial showed osimertinib plus bevacizumab failed to provide any PFS benefit over osimertinib monotherapy as a front-line treatment for *EGFR* mutated patients with brain metastasis.^32^

As we know, monitoring the dynamic changes of mutation load in peripheral blood can serve as an effective indicator of treatment efficacy. But the impact of EGFR TKI and anti-angiogenic therapy on TML remains undefined. Our study found no notable difference in TML at baseline and the first posttreatment efficacy evaluation. But there is an apparently greater reduction in the rates of total *EGFR* and *EGFR* ex21L858R treated with gefitinib plus anlotinib at the first posttreatment efficacy evaluation. The lower mutation rate of key driver genes indicates stronger tumor inhibition and anti-tumor activity. Meanwhile, upon disease progression, the TML of patients receiving gefitinib plus placebo was significantly higher than patients receiving gefitinib plus anlotinib, suggesting that TML rises coincident with disease progression. Dual therapy with an EGFR TKI and an anti-angiogenic agent suppresses TML to a greater extent than EGFR TKI monotherapy. Similar change trajectories were observed in *EGFR* ex21L858R, ex19del and amplification. A similar study had also proved that dual therapy with ramucirumab and erlotinib could suppress *EGFR*-activating mutation allele count in *EGFR-*mutated, advanced NSCLC.^40^ Tracking temporal changes in TML and the frequencies of key driver gene mutations in individual patients could better delineate the dynamics of gene mutations during disease progression. Consistent with other studies, mutated TP53 was enriched in *EGFR*-mutated NSCLC, occurring in 57.8% of the patients at baseline, which falls within the reported range of 54.6%%-64.6% for mutated *TP53*.^41–43^ The presence of mutated *TP53* is reported to be an adverse predictor of TKI therapeutic outcome in NSCLC.^26^ In this study, dual inhibition likely contributed to the reduction in the occurrence of *TP53* mutations, resulting in a lower rate of mutated *TP53* at the first posttreatment efficacy evaluation and at the time of disease progression. Above all, our study further showed that dual inhibition of VEGF and EGFR signaling pathways was more effective in suppressing the TML and the frequencies of mutated key driver genes than TKI monotherapy.

Currently, there are no established enough molecular biomarkers that can reliably predict clinical response or resistance to anti-angiogenic agents, although patients with *EGFR* ex21L858R or baseline brain metastasis derive more benefits from dual inhibition in ARTEMIS-CTONG1509 study.^18^ We observed the trajectory of TML changes and further evaluated TML as a biomarker to explore survival benefit associated with the dual inhibition. Anlotinib added to gefitinib led to a 6.2-month extension in PFS compared to gefitinib alone, with a 62% reduction in the risk of progression in patients with high TML, proving that patients with high TML could benefit more from gefitinib plus anlotinib. The presence of CNV of resistance-related genes including *EGFR* was associated with a poorer response to osimertinib in advanced *EGFR*-mutated lung adenocarcinoma patients.^28^ The current study found that anlotinib added to gefitinib led to a 4.9-month extension in PFS compared to gefitinib alone, with a 73% reduction in the risk of progression. In short, except for patients with brain metastases, those with high TML or *EGFR* amplification gain more benefits from combined TKI therapy and anti-angiogenic therapy.

The current study excluded patients with *EGFR*^*T790M*^-mutated NSCLC at baseline. Upon disease progression, the rate of secondary *EGFR*^*T790M*^-mutation rose to 38% in patients on gefitinib plus placebo and 17% in patients treated with gefitinib plus anlotinib. This is lower than 40–50% in patients who develop secondary *EGFR*^*T790M*^ mutations as a result of treatment with first/second-generation EGFR TKIs.^44^ The rate of *EGFR*^*T790M*^ mutations is related to tumor burden and TML. The low rate of this mutation in our patients suggests that gefitinib plus anlotinib was more effective in reducing tumor burden and TML. In this study, mutations were detected using peripheral blood samples, which tend to be less frequently detected in carcinoma tissues, which may partially explain the low rate of *EGFR*^*T790M*^ mutations in our patients.

The toxicity profile of gefitinib plus anlotinib is consistent with that of gefitinib and anlotinib monotherapy. Rash and diarrhea are common in patients treated with gefitinib^5^; any grade diarrhea and rash occurred in 65.8% and 65.2% of the patients receiving gefitinib plus anlotinib *versus* 39.0% and 52.6% of those receiving gefitinib plus placebo, respectively. The rate of liver abnormalities is reportedly higher with gefitinib in Asian patients than non-Asian patients^45^ and ALT and AST elevations occurred in 48.4% of our patients in both groups. Hepatic impairment can be resolved by dose reductions or treatment interruptions. Grade 3 or higher hypertension (28.4% *vs*. 5.2%) and proteinuria (1.9% *vs*. 0%), two main AEs of anti-VEGF therapy, were more frequent in patients receiving gefitinib plus anlotinib than patients receiving gefitinib plus placebo and are largely consistent with those of anlotinib therapy for other tumors.^46^ The rate of grade 3 or higher hypertension and proteinuria is lower than that (hypertension 37–60% and proteinuria 7–8%) reported for erlotinib plus bevacizumab in other trials.^38,47–49^ Overall, gefitinib plus anlotinib had a manageable toxicity profile in advanced NSCLC patients.

The major limitations should be addressed in this study. Third generation EGFR-TKIs, which are being increasingly employed in the treatment of NSCLC, were not investigated in the current trial. At the time the current trial was started (April 2019), osimertinib was not available in China. The agent was approved in September 2019 in China as first line treatment for *EGFR* mutated NSCLC.^50^ It remains to be investigated whether anlotinib added to a third-generation EGFR-TKI would confer similar or greater benefit on *EGFR*-mutated, advanced NSCLC patients. The study enrolled mostly Han Chinese patients, and it still needs to be explored whether the study findings are applicable to non-Asian patients. Though the study findings suggest that high TML and EGFR gain appeared to be potential predictors for anlotinib benefit. However, given the small sample size in these subgroups in this study, the findings remain exploratory. This benefit with regards to objective response was observed in advanced NSCLC patients with high TML treated with anlotinib plus immunotherapy (≥10 Muts/Mb 85.7% *vs*. <10 Muts/Mb 63.6%) in a phase 1 study^24^ and was also reported in pretreated advanced biliary tract cancer patients.^51^ However, the mechanism whereby anlotinib favors patients with high genomic instability and the prognostic significance of TML and EGFR gain remain to be investigated.

In conclusion, the combination of EGFR TKI with gefitinib and anti-angiogenic therapy with anlotinib significantly improved PFS with a manageable safety profile for patients with untreated advanced NSCLC harboring *EGFR* activating mutations. Particularly, patients with brain metastases, those with high TML, and patients harboring *EGFR* amplification gain more benefits from gefitinib plus anlotinib compared with gefitinib monotherapy. The findings support further investigation of the third generation TKI plus antiangiogenic agent in advanced stage trials in the frontline and later line setting for advanced NSCLC patients with *EGFR* activating mutations. Studies on anlotinib plus third generation TKIs for advanced NSCLC are currently ongoing (NCT04770688; NCT06043973).

## Materials and methods

### Ethics approval and consent to participate

The trial protocols were approved by the independent ethics committee at each participating center and complied with the International Ethical Guidelines for Biomedical Research Involving Human Subjects. The studies were conducted according to the International Conference on Harmonisation guidelines for Good Clinical Practice and the Declaration of Helsinki. All patients provided written informed consent prior to the study. This report followed the Consolidated Standards of Reporting Trials (CONSORT) reporting guideline. The trial is registered with ClinicalTrials.gov (NCT04028778).

### Study design and participants

FL-ALTER, a multicenter, randomized, double-blind, phase III trial, was conducted at 18 hospitals (Supplementary Appendix I) in the People’s Republic of China. It enrolled adult patients (aged above 18 years) with histologically confirmed American Joint Committee on Cancer (AJCC) stage IIIB or IV NSCLC. Patients with an ex19del or ex21L858R *EGFR* mutation were eligible. Patients had received no prior chemotherapy or targeted therapy. They should have at least one measurable lesion per Response Evaluation Criteria in Solid Tumors (RECIST) version^52^ 1.1, adequate organ function, and an Eastern Cooperative Oncology Group (ECOG) performance status score of 0 or 1. We excluded patients with *EGFR* T790M-mutated NSCLC. Patients were ineligible if they had hypertension requiring at least 2 types of antihypertensive medications, prior or current interstitial lung disease, and abdominal fistulation, gastrointestinal perforation, or abdominal abscess in the preceding 6 months before enrollment. Patients with symptomatic, unstable brain metastases, arterial or venous embolism within 12 months of enrollment, or at a higher risk of bleeding were also excluded. Full eligibility criteria are provided in the study protocol (Data Supplement).

### Randomization and treatments

The participants were randomized in a 1:1 ratio to treatment with gefitinib plus anlotinib or gefitinib plus placebo using the Interactive Web Response System (IWRS). Randomization was stratified by sex (male *vs*. female), ECOG performance status (0 *vs*. 1), *EGFR* mutation subtype (ex19del *vs*. ex21L858R) and pathologic types (adenocarcinoma *vs*. others). Both patients and investigators were blinded to treatment allocation.

Gefitinib 250 mg and anlotinib 12 mg (Chia Tai Tianqing Pharmaceutical Group Co.) were taken orally once daily on days 1–14 per cycle in a two weeks on, one week off schedule, which lasted for 3 weeks. Anlotinib treatment could be interrupted due to toxicities but should be resumed at the same or a lower dose level. Anlotinib treatment was discontinued if patients experienced severe toxicities (gastrointestinal perforation [any grade], arterial thromboembolism [any grade], venous thromboembolism [grade 4], hypertensive crisis or cerebral hemorrhage [any grade], leukoencephalopathy syndrome [any grade], pulmonary hemorrhage [grade ≥2] or other hemorrhages [grade ≥3], and renal, hepatic, cardiac or neurologic toxic effects [grade 4]). Treatment was also discontinued if patients had not recovered from a toxic effect requiring treatment interruptions (hematologic or nonhematologic toxicities [grade ≥3]). Two levels of dose reduction (12 mg/d to 10 mg/d and 10 mg/d to 8 mg/d) were allowed for anlotinib and treatment with anlotinib was discontinued if more than two levels of dose reduction were required. Patients who were intolerant of anlotinib were permitted to continue gefitinib treatment. Gefitinib treatment was discontinued upon acute onset or aggravation of respiratory symptoms, new onset ocular symptoms or diagnosis of interstitial lung disease. Dose reductions of gefitinib were not allowed. They were allowed to continue anlotinib treatment at the discretion of investigators. Both drugs were continued until disease progression, intolerable toxicities, death, withdrawal of consent or termination at the discretion of investigators. Although treatment should be terminated upon occurrence of progressive disease (PD) per the study protocol, patients were allowed to continue the study treatment beyond radiological progression when deemed clinically beneficial by the investigators.

Patients were allowed to receive bisphosphonates for bone metastasis and palliative radiotherapy was allowed for uncontrollable metastasis-associated pain with the irradiation field confined to less than 5% of the bone marrow.

### Assessments and outcomes

Responses were evaluated radiologically by investigators per RECIST v1.1 at baseline, at the end of each cycle for the first 4 cycles, and the end of every 2 cycles from cycle 5 until disease progression, intolerable toxicities, death, or withdrawal of consent. Complete (CR) and partial response (PR) had to be confirmed radiologically at least 4 weeks later and SD at least 8 weeks after an initial response. Treatment-emergent adverse events (TEAEs) were evaluated throughout the treatment period and to 30 days post the final dose using the Common Toxicity Standards of the National Cancer Institute (NCI CTC AE) version 4.0.

The primary endpoint was PFS (time from randomization to PD or death of any cause, whichever occurred earlier). The secondary endpoints included OS (time from randomization to death of any cause), objective response rate (proportion of patients who achieved CR or PR), disease control rate (proportion of patients who achieved CR, PR, or SD), duration of response (time from the first documented CR or PR to the first documented disease progression or relapse), and time from randomization to radiological progression.

### Next generation sequencing

Blood samples for ctDNA analysis were collected from each patient at baseline, first evaluation, and PD, and transferred to Clinical Laboratory Improvement Amendments (CLIA)-certified/College of American Pathologists (CAP)-accredited laboratory of OrigiMed Co., Ltd. (Shanghai, China) for plasma extraction and genomic testing. CtDNA were prepared using the QIAGEN QIAamp Circulating Nucleic Axid Kit according to the manufacturer’s instructions.

Ten mL peripheral blood was withdrawn before treatment, at the first efficacy evaluation for detection of the effect of ctDNA clearance on efficacy and dynamic monitoring and upon documentation of PD to evaluate mechanisms of resistance. Dynamic detection of plasma ctDNA was performed using the NGS-based Qiyuan^TM^ 329-gene panel (OrigiMed, Shanghai, China). Circulating tumor DNA (ctDNA) analysis was performed on the basis of read depth, which was first normalized to sequencing depth and corrected for GC content to reduce technical bias. Subsequently, the relative change in ctDNA samples was quantified as a ratio using a baseline established from normal human cfDNA libraries and a log2 transformation was performed to obtain a log2 ratio for further analysis. The log2ratio was then segmented using the cyclic binary segmentation (CBS) algorithm to determine the log2 ratios of discrete fragments. The change in copy number for these segments was inferred as 2^(1 + log2ratio). The copy number ≥2.5 indicates gene amplifications whereas the copy number ≤1.2 indicates gene deletions. Genes were captured and sequenced at a mean depth of approximately 15,000× using an Novaseq 6000 (Illumina, CA, USA), followed by noise filtering and molecular tracking, and variant calling for single nucleotide variants (SNVs). Sequencing files were analyzed using Origimed’s internal bioinformatic pipelines to identify SNVs using MuTect (v1.17), copy number alterations (CNAs) using Control-FREEC (v9.7), insertion-deletion (indels) using PINDEL (v2.05), and gene fusion events. By comparing the ctDNA with matched white blood cell samples, germline mutations were filtered out and retained, and only somatic mutations were identified in the plasma samples during the analysis. Somatic mutations with the variant allelic fraction (VAF) were analyzed for tracking dynamic ctDNA levels over time relative to baseline. To enable relative change calculations between timepoints, if a variant was not detectable, the VAF percentage (VAF%) was set to the assay’s lowest detection limit of 0.2%. The functional impact of each genomic alteration was annotated using SnpEff3.0. The results were annotated to several databases, including the Reference Sequence (RefSeq), 1000 Genomes, Genome Aggregation Database (gnomAD), the Exome Aggregation Consortium (ExAC), NHLBI GO Exome Sequencing Project 6500 (ESP6500), Sorting Intolerant from Tolerant (SIFT), PolyPhen, and Catalogue of Somatic Mutations in Cancer (COSMIC) databases.

In addition, TML was estimated by dividing the total number of mutations by the total length of the coding sequence (CDS) region. Mutations were defined by the following criteria: (1) SNVs or insertions/deletions were located within the CDS region and (2) the mutation had an abundance of ≥0.25%.

### Statistical analysis

We hypothesized that the PFS of patients treated with gefitinib plus anlotinib would be superior to that of patients treated with gefitinib plus placebo. Based on previous studies,^5,30,53–57^ we assumed a median PFS of 10 months for the gefitinib plus placebo group and 15 months for the gefitinib plus anlotinib group. A 5-month increase in the median PFS at 15 months was considered to be clinically meaningful to show superiority of gefitinib plus anlotinib over gefitinib plus placebo. To detect this improvement in PFS with 80% power at a two-sided 5% significance level, 192 events (88 with gefitinib plus anlotinib and 104 with gefitinib plus placebo) were required. Assuming an attrition rate of 20%, a sample size of 310 was planned.

All statistical analyses were prespecified and followed the intention-to-treat (ITT) principle and were conducted using SAS version 9.3 or above (SAS Institute Inc., Cary, NC, USA). An independent data monitoring committee (IDMC) carried out interim analyses. The Full Analysis Set (FAS) included all patients who were randomized and received at least one dose of the study medications and the Per Protocol Set (PPS) included all patients who were randomized and received at least one dose of the study medications and had at least one post treatment radiological evaluation. Efficacy analysis was based on the FAS. A Cox regression model was used to estimate HR stratified by sex, ECOG performance status, *EGFR* mutation subtype and pathologic types and its 95% CI. PFS, OS, time to PD, and duration of response and their 95% CI were estimated using the Kaplan–Meier method and compared using log-rank test. The ORR and DCR and their 95% CI were compared using Cochran-Mantel-Haenszel test or *χ*^2^ test. No imputation was done for missing data.

The Safety Set included all patients who had received at least one dose of the study medications and had post treatment safety data. AEs were mainly analyzed using descriptive statistics.

All tests were two-tailed with a level of significance set at *P* ≤ 0.05.

### Supplementary information

supplementary files

Clinical Study Protocol

## Data Availability

The raw sequencing data reported in this paper have been deposited in the Genome Sequence Archive in National Genomics Data Center, China National Center for Bioinformation/Beijing Institute of Genomics, Chinese Academy of Sciences (https://ngdc.cncb.ac.cn/gsa-human). These data are accessible under the accession number: HRA007966. These data are under controlled access by human privacy regulations and are only available for research purposes. Access to the data can be granted following approval from the Data Access Committee of the GSA-human database. And other datasets used and/or analyzed during the current study are also available from the corresponding author on reasonable request.
