# Supplementary material for: Gefitinib (an EGFR tyrosine kinase inhibitor) plus anlotinib (an multikinase inhibitor) for untreated, EGFR-mutated, advanced non-small cell lung cancer (FL-ALTER): a multicenter phase III trial
Source: Signal Transduct Target Ther. 2024 Aug 13;9:215. doi: 10.1038/s41392-024-01927-9 (PMC11319491; doi:10.1038/s41392-024-01927-9)
Supplement: Supplementary file 1 — supplementary files [file 41392_2024_1927_MOESM1_ESM.docx]

Supplementary Materials for

**Gefitinib plus anlotinib for untreated, *EGFR-*mutated, advanced NSCLC (FL-ALTER): A multicenter phase III trial**

Hua-qiang Zhou^1^†, Ya-xiong Zhang^1^†, Gang Chen^1^†, Qi-Tao Yu^2^†，Hua Zhang^3^†, Guo-Wu Wu^4^, Di Wu^5^, Ying-Cheng Lin^6^, Jun-Fei Zhu^7^, Jian-Hua Chen^8^, Xiao-Hua Hu^9^, Bin Lan^10^, Ze-Qiang Zhou^11^, Hai-Feng Lin^12^, Zi-Bing Wang^13^, Xiao-Lin Lei^14^,Suo-Ming Pan^15^,Li-Ming Chen^16^, Jian Zhang^17^, Tian-Dong Kong^18^, Ji-cheng Yao^19^, Xin Zheng^19^, Feng Li^19^, Li Zhang^1*^ ,Wen-Feng Fang^1*^

Correspondence to: Wen-Feng Fang: [fangwf@sysucc.org.cn](mailto:fangwf@sysucc.org.cn) Li Zhang: [zhangli@sysucc.org.cn](mailto:zhangli@sysucc.org.cn)

**This file includes:** Supplementary Figures S1 to S4; Supplementary Tables S1 to S2;

Clinical Study Protocol


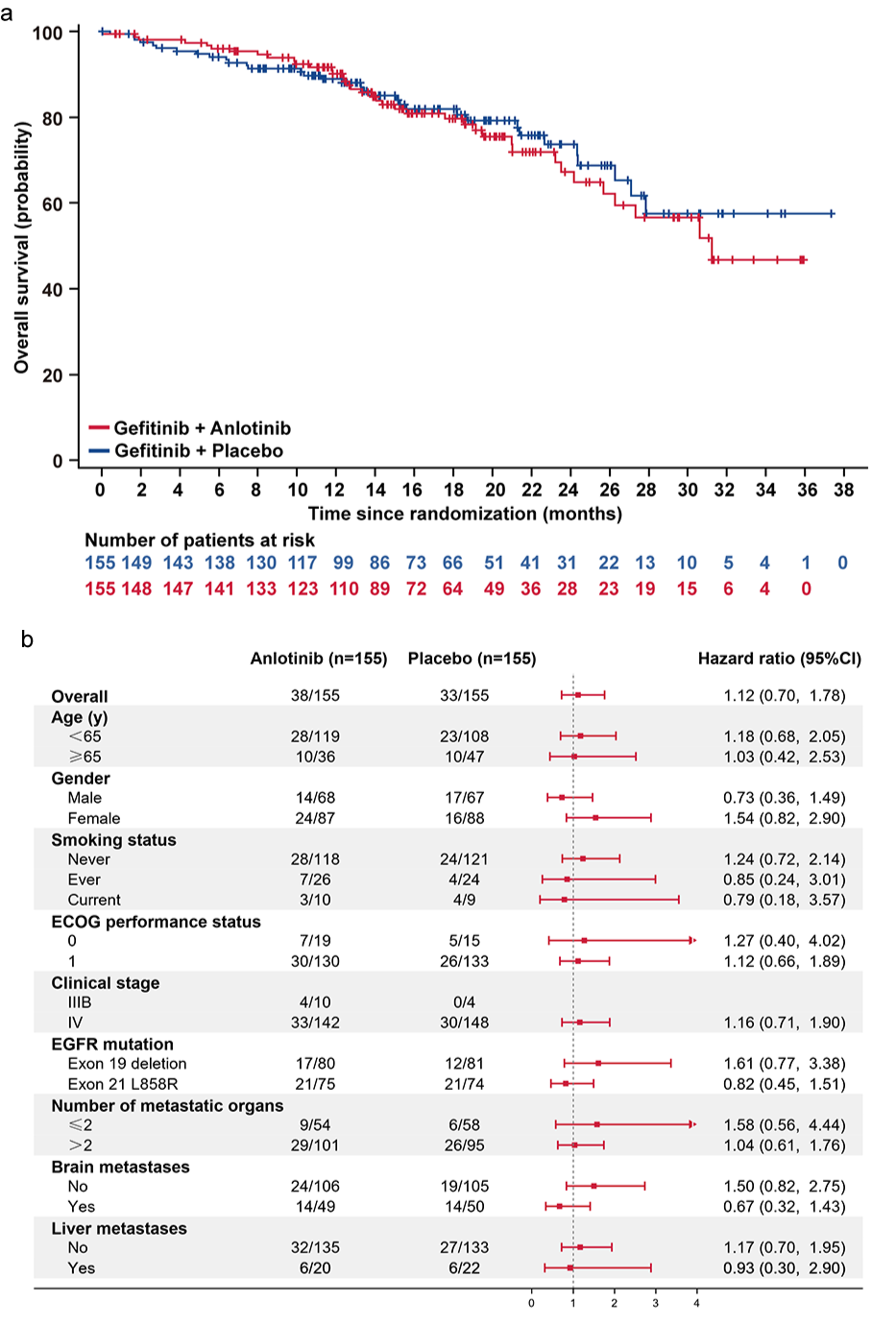


**Supplementary Figure 1** Gefitinib plus anlotinib does not improve overall survival (OS) of advanced NSCLC patients. **a** The Kaplan-Meier curves of OS of advanced NSCLC patients treated with gefitinib plus anlotinib or gefitinib plus placebo. **b** Forest plots for OS.


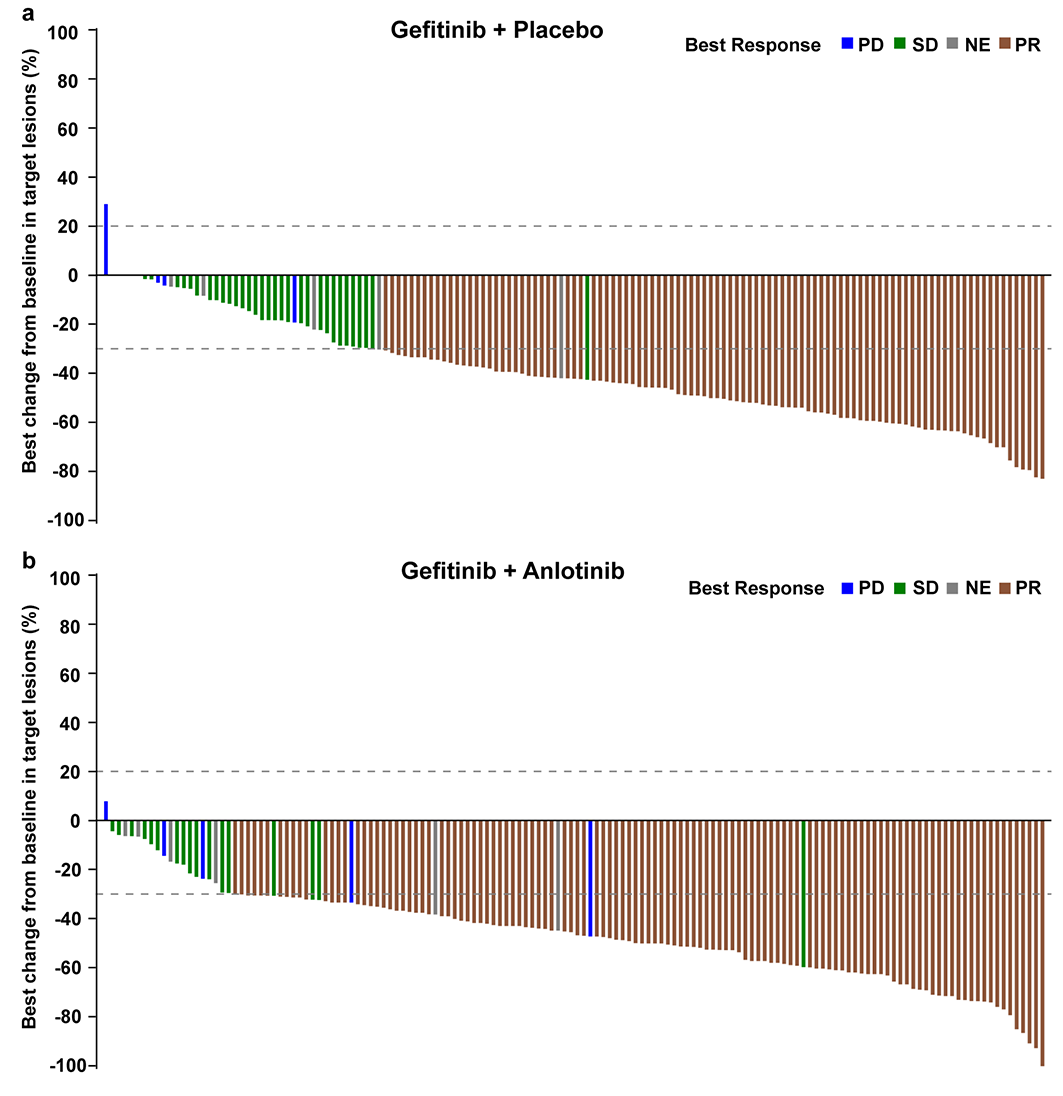


**Supplementary Figure 2** Waterfall plots of the best percentage changes for the sum of target lesion diameters are shown for individual patients treated with gefitinib plus anlotinib **a** or gefitinib plus placebo **b** as assessed per RECIST v1.1. The green dotted line indicates a 30% reduction, and the orange dotted line indicates a 20% increase in the target lesion size. Each bar represents one patient in efficacy evaluable patients.


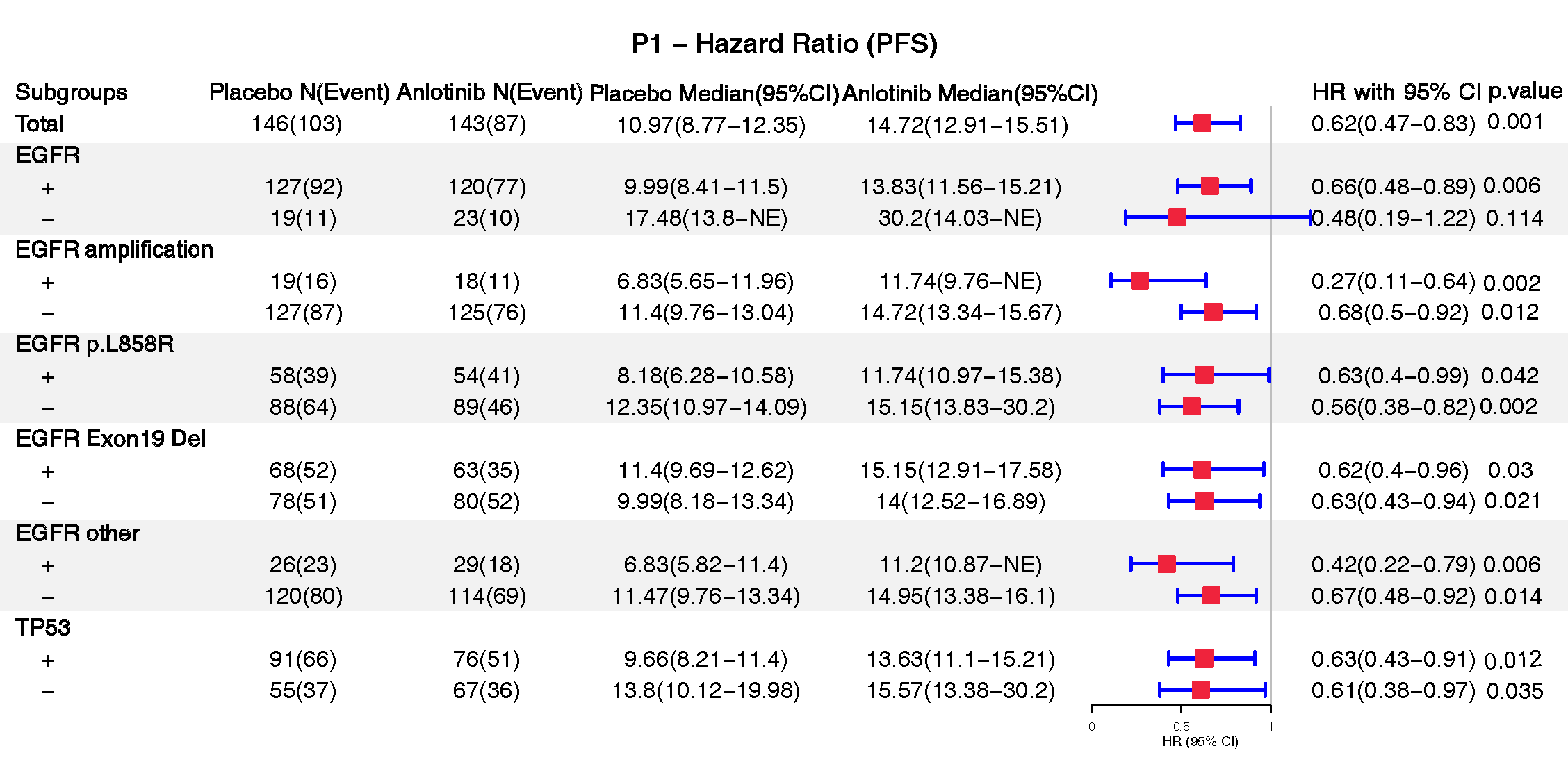


**Supplementary Figure 3** Forest plots for PFS of patients with *EGFR* or TP53 mutations based on ctDNA status. *EGFR*_other is a summary of all mutation types except for pL858R,_exon19_del, and p790M.


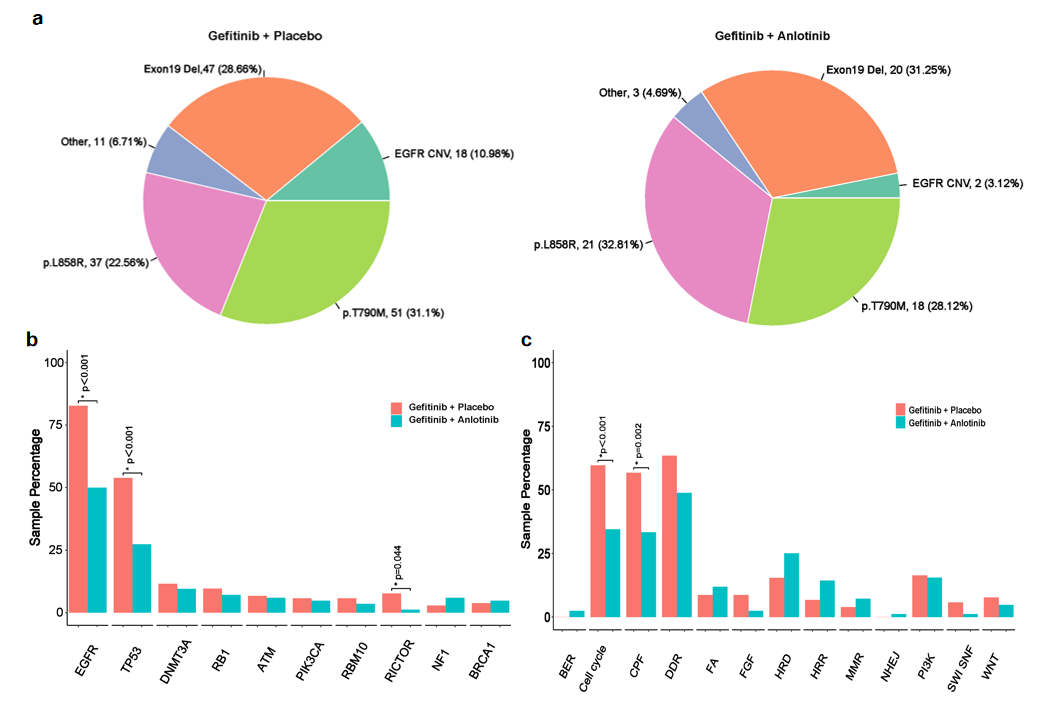


**Supplementary Figure 4** *EGFR* mutations and the associated gene pathways at PD. **a** Rates of secondary *EGFR* mutations upon disease progression. **b** Rates of mutated genes at the time of disease progression. **c** Gene pathway analysis shows involved signaling pathways. BER:Base excision repair, Cell Cycle: Cel Cycle, CPF: Cleavage and polyadenylation factor, DDR:DNA Damage Response , FA:Fanconi Anemia, FGF:Fibroblast Growth Factor, HRD:Homologous Recombination Deficiency, HRR:Homologous Recombination Deficiency, MMR:Mismatch Repair, NHEJ:Non-homologous End Joining, PI3K: phosphoinositide 3-kinase, SWI.SNF:SWItch/Sucrose Non-Fermentable, WNT:Wingless-Type MMTV Integration Site Family

**Supplementary table 1 Efficacy measures**

|  | Gefitinib plus anlotinib | Gefitinib plus placebo | *P* |
| --- | --- | --- | --- |
| Variable | N=155 | N=155 |  |
| Survival outcomes-FAS |  |  |  |
| Progression-free survival |  |  |  |
| Median (95% CI) | 14.8 (12.9-15.4) | 11.2 (9.6-12.2) | 0.003^#^ |
| 12 months, % (95% CI) | 62.2 (53.6-69.7) | 42.2 (33.6-50.6) |  |
| 24 months, % (95% CI) | 24.0 (15.5-33.6) | 12.2 (6.4-20.0) |  |
| Overall survival |  |  |  |
| Median (95% CI) | 31.2 (25.7-NE) | NR (27.1-NE) | 0.644* |
| 12 months, % (95% CI) | 90.1 (83.8-94.0) | 88.9 (82.4-93.1) |  |
| 24 months, % (95% CI) | 67.2 (55.3-76.6) | 73.6 (62.9-81.7) |  |
| Tumor response-FAS |  |  |  |
| Best objective response, no. (%) |  |  |  |
| Complete response | 0 | 0 |  |
| Partial response | 118(76.1) | 100(64.5) |  |
| Stable disease | 17(11.0) | 35(22.6) |  |
| Progressive disease | 5 (3.2) | 5 (3.2) |  |
| Not evaluable | 15 (9.7) | 15 (9.7) |  |
| Objective response rate, % (95% CI) | 76.1(68.6-82.6) | 64.5(56.4-72.0) | 0.025**^&^** |
| Disease control rate, % (95% CI) | 87.1(80.8-91.9) | 87.1(80.8-91.9) | > 0.999**^&^** |
| Median (95% CI) duration of response, months | 12.5(11.1-16.2) | 9.5(7.0-10.3) | < 0.001* |
| CI, confidence interval; FAS, Full Analysis Set; NE, not evaluable; NR, not reached.  Responses were evaluated per RECIST version 1.1. Progressive-free survival and overall survival were calculated using the Kaplan-Meier method.  ^#^Stratified log-rank test; *log-rank test; ^&^chi-square test | | | |

**Supplementary table 2 Subsequent antitumor therapies in the study patients**

| Subsequent antitumor therapies | The anlotinib group(n=44) | The placebo group (n=63) |
| --- | --- | --- |
| Osimertinib | 13(29%) | 26(41%) |
| **Almonertinib** | 17(39%) | 21(33%) |
| Furmonertinib | 0 | 2(3%) |
| Gefitinib | 9(20%) | 3(5%) |
| Icotinib | 1(2%) | 0 |
| Afatinib | 0 | 1(1.5%) |
| Platinum-containing chemotherapy | 2(4.5%) | 6(10%) |
| Platinum-containing chemotherapy plus bevacizumab | 2(4.5%) | 2(3%) |
| Others (herbal medicine) | 0 | 2(3%) |
