# Supplementary material for: Gefitinib (an EGFR tyrosine kinase inhibitor) plus anlotinib (an multikinase inhibitor) for untreated, EGFR-mutated, advanced non-small cell lung cancer (FL-ALTER): a multicenter phase III trial
Source: Signal Transduct Target Ther. 2024 Aug 13;9:215. doi: 10.1038/s41392-024-01927-9 (PMC11319491; doi:10.1038/s41392-024-01927-9)
Supplement: Supplementary file 2 — Clinical Study Protocol [file 41392_2024_1927_MOESM2_ESM.docx]

**A phase III multi-center, randomized, double-blind, parallel controlled trial of anlotinib plus gefitinib versus placebo plus gefitinib as first-line therapy in stage IIIB-IV EGFR-mutated non-small cell lung cancer (NSCLC)**

**Clinical Study Protocol**

**Protocol Number: FL-ALTER**

**Version Number:** 1.6

**Version Date:** October 29, 2021

**Unit of Data Management:** Sun Yat-sen University Cancer Center

**Sponsor:** Sun Yat-sen University Cancer Center

**Protocol Signing Page**

**Signature of the Investigator**

I will conscientiously perform my duties as an investigator according to guidelines for Good Clinical Practice (GCP) in China, and participate in or directly guide this clinical study. We have read and confirmed the protocol (protocol number: FL-ALTER; version number: 1.6; version date: October 29, 2021). I agree to perform my duties in accordance with Chinese law, the Declaration of Helsinki, the Chinese GCP, and the study protocol. I will make modifications to the protocol only after informing the sponsor and with the approval of the ethics committee unless measures are necessary to protect the safety, rights, and interests of the subjects.

| **Unit of Clinical Study:** Sun Yat-sen University Cancer Center | | |
| --- | --- | --- |
|  | | |
|  |  |  |
| Principal Investigator (print form) | Principal Investigator (signature) | Signature Date (year/month/day) |

**Protocol Signing Page**

**Signature of the Sponsor**

We have read and confirmed the protocol (protocol number: FL-ALTER; version number: 1.6; version date: October 29, 2021). I agree to perform my duties in accordance with Chinese law, the Declaration of Helsinki, the Chinese GCP, and the study protocol.

| **Sponsor:** Sun Yat-sen University Cancer Center | | |
| --- | --- | --- |
|  | | |
|  |  |  |
| Principal Head (print form) | Principal Head (signature) | Signature Date (year/month/day) |

**Contents**

[Protocol synopsis 5](#_Toc124944034)

[List of abbreviations 10](#_Toc124944035)

[1. Background 13](#_Toc124944036)

[1.1 Overview of lung cancer and targeted therapy 13](#_Toc124944037)

[1.2 Drug information 16](#_Toc124944038)

[1.3 Pharmacological study of anlotinib 16](#_Toc124944039)

[1.4 Toxicology of anlotinib 16](#_Toc124944040)

[1.5 Pharmacokinetics of anlotinib 17](#_Toc124944041)

[1.6 Clinical study 24](#_Toc124944042)

[2. Study Objective 24](#_Toc124944043)

[3. Study Design 25](#_Toc124944044)

[3.1 Study design 25](#_Toc124944045)

[3.2 Type of comparison 25](#_Toc124944046)

[3.3 Estimating sample size 25](#_Toc124944047)

[4. Randomization and Blinding 27](#_Toc124944048)

[4.1 Randomization design 27](#_Toc124944049)

[4.2 Blinding design 27](#_Toc124944050)

[4.3 Handling of randomization errors 27](#_Toc124944051)

[5. Subjects 27](#_Toc124944052)

[5.1 Number of subjects 27](#_Toc124944053)

[5.2 Inclusion criteria 27](#_Toc124944054)

[5.3 Exclusion criteria 29](#_Toc124944055)

[5.4 Dropout/removal criteria 30](#_Toc124944056)

[5.5 Termination criterion 30](#_Toc124944057)

[6. Study Drugs 30](#_Toc124944058)

[6.1 Overview of drugs 30](#_Toc124944059)

[6.2 Dose and administration regimens 31](#_Toc124944060)

[6.3 Drug administration, distribution, and recovery 32](#_Toc124944061)

[6.4 Destruction of study drugs 32](#_Toc124944062)

[7. Dose Adjustment and Discontinuation 32](#_Toc124944063)

[7.1 Dose adjustment of anlotinib 32](#_Toc124944064)

[7.2 Dosage suspension and reduction 32](#_Toc124944065)

[7.3 Dose adjustment of gefitinib 34](#_Toc124944066)

[7.4 Management of anlotinib-related AEs 34](#_Toc124944067)

[8. Concomitant Medications 39](#_Toc124944068)

[8.1 Drugs prohibited or used with caution during the study 39](#_Toc124944069)

[8.2 Permitted concomitant medications/treatments 40](#_Toc124944070)

[9. Study Procedures 40](#_Toc124944071)

[9.1 Screening 40](#_Toc124944072)

[9.2 Treatment period 42](#_Toc124944073)

[9.3 End-of-treatment and withdrawal study 44](#_Toc124944074)

[9.4 30-day post-withdrawal follow-up 44](#_Toc124944075)

[9.5 Follow-up of survival 45](#_Toc124944076)

[9.6 Unplanned follow-up 45](#_Toc124944077)

[10. Study Assessments 45](#_Toc124944078)

[10.1 Efficacy assessment 45](#_Toc124944079)

[10.2 Safety analysis 46](#_Toc124944080)

[10.3 Drug resistance mechanisms 50](#_Toc124944081)

[11. Ethical Considerations and Informed Consent 50](#_Toc124944082)

[11.1 Ethical considerations 50](#_Toc124944083)

[11.2 Informed consent 50](#_Toc124944084)

[12. Sponsor/Investigator Responsibilities 51](#_Toc124944085)

[12.1 Sponsor 51](#_Toc124944086)

[12.2 Investigator 51](#_Toc124944087)

[13. Data Management 51](#_Toc124944088)

[13.1 Data collection 51](#_Toc124944089)

[13.2 Statistical analysis 52](#_Toc124944090)

[13.3 Interim analysis 53](#_Toc124944091)

[13.4 Data storage 53](#_Toc124944092)

[13.5 Publication policy 53](#_Toc124944093)

[References 54](#_Toc124944094)

**Protocol synopsis**

| Study Drug | Anlotinib hydrochloride capsule |
| --- | --- |
| Protocol Title | A phase III multi-center, randomized, double-blind, parallel controlled trial of anlotinib plus gefitinib versus placebo plus gefitinib as first-line therapy in stage IIIB-IV EGFR-mutated non-small cell lung cancer (NSCLC) |
| Protocol Number | FL-ALTER |
| Version Number | 1.6 |
| Version Date | October 29, 2021 |
| Sponsor | Sun Yat-sen University Cancer Center |
| Study Design | A multi-center, randomized, double-blind, placebo-controlled parallel study |
| Study Nature | An investigator-initiated clinical study |
| Study Population | Treat-naive patients with stage IIIB-IV EGFR-mutated NSCLC |
| Sample Size | A total of 310 patients were randomized in a 1:1 ratio to the experimental group and the control group. |
| Objective | To observe and evaluate the efficacy and safety of anlotinib combined with gefitinib in patients with advanced EGFR-mutated NSCLC. |
| Inclusion Criteria | Subjects can participate in the study only if all the following criteria are met:   1. Age 18-75 years; 2. Eastern Cooperative Oncology Group performance status (ECOG PS) of 0-1; 3. Predicted life expectancy of ≥12 weeks; 4. Pathologically confirmed diagnosis of stage IIIB (not suitable for radiotherapy)/IV NSCLC; at least one measurable lesion based on RECIST v1.1 (tumor lesions with the longest diameter ≥10mm on CT scan, and lymph nodes with a short diameter ≥15mm on CT scan; no local treatment such as radiotherapy and freezing for measurable lesions); 5. Patients with primary NSCLC harboring activating EGFR mutations (exon 19 deletions or 21 L858R point mutation) according to any validated method; 6. No previous chemotherapy or other targeted therapy;   Note: Treatments during neoadjuvant therapy are not considered in treatment regimens; patients relapsing within six months of the completion of neoadjuvant therapy (partially defined as first-line treatment) could not be enrolled in this study; if recurrence occurs after more than 6 months, neoadjuvant therapy is not included in the treatment regimens.   1. Patients who have previously received radiotherapy are eligible, with radiation therapy to <25% of bone marrow (Cristy and Eckerman 1987) and without full pelvic or chest irradiation; prior radiation therapy must have been completed at least 4 weeks before enrollment and acute toxicity induced by radiation therapy must have recovered; local lesions that were treated with radiation could not be included in the measurable lesions unless significant progression of the lesion was recorded after the last radiotherapy; 2. Adequate function of the important organs as evidenced by the following:    - - 1. Hemanalysis (no blood transfusion within 14 days, no correction with G-CSF or other hematopoietic stimulation factors): 3. absolute neutrophil count (ANC) ≥1.5×10^9^/L； 4. platelets (PLT) ≥100×10^9^/L； 5. hemoglobin (Hb) ≥100g/L；    - - 1. Biochemistry    1. total bilirubin (TBIL) <1.5×ULN； 6. alanine aminotransferase (ALT) and aspartate aminotransferase (AST) <2.5×ULN; ALT and AST <5×ULN in patients with liver metastases; 7. blood Urea Nitrogen (BUN) and creatinine (Cr) ≤1×ULN or creatinine clearance rate ≥50ml/min (Cockcroft-Gault formula); 8. Women of reproductive age must have already used effective contraception or have negative serum or urine pregnancy test results within 7 days before enrollment and be willing to use an adequate method of contraception during the trial period and 8 weeks after the last drug administration. For men, consent is given to use an appropriate method of contraception or to have been surgically sterilized during the trial period and 8 weeks after the last drug administration; 9. Be willing and able to provide written informed consent for the trial, and comply with all aspects of the protocol. |
| Exclusion Criteria | Subjects who meet any of the following criteria are not eligible to enter the study:   - 1. Small cell lung cancer (including mixed small cell and non-small cell lung cancer);   2. Symptomatic brain metastases (patients with stable brain metastases who have completed treatment 21 days before enrollment are eligible, but have no symptoms of cerebral hemorrhage confirmed by brain MRI, CT, or venography);   3. Tumor lesion of ≤5 mm from the large blood vessels, or central tumor invading the local large blood vessels, or significant pulmonary cavitary or necrotic tumors by imaging (computed tomography [CT]/ magnetic resonance imaging [MRI]);   4. Patients with hypertension who are being treated with a combination of two or more antihypertensive drugs;   5. Patients with positive T790M mutations from genetic test;   6. Cardiovascular diseases: class II or more of myocardial ischemia or myocardial infarction, arrhythmia due to poorly controlled (including men with corrected QT interval [QTc] ≥450 ms and women with QTc ≥470 ms); patients in New York Heart Association (NYHA) class III-IV cardiac dysfunction, or left ventricular ejection fraction (LVEF) < 50% by echocardiography;   7. Patients with a history of interstitial lung disease or concurrent interstitial lung disease;   8. Abnormal coagulation function (INR >1.5 or prothrombin time [PT] >ULN+4 seconds or APTT >1.5 ULN) with a bleeding tendency, or are receiving thrombolytic or anticoagulant therapy;   9. Hemoptysis (2 teaspoons or more per day) before enrollment;   10. Clinically significant bleeding symptoms or a definite bleeding tendency within 3 months before enrollment, such as gastrointestinal bleeding, bleeding hemorrhoids, gastric hemorrhagic ulcers, baseline levels for fecal occult blood (++ or above), or vasculitis;   11. Arterial and venous thrombosis within 12 months prior to enrollment, such as cerebrovascular accidents (including temporary ischemic attacks, cerebral hemorrhage, and cerebral infarction), deep vein thrombosis, and pulmonary embolism;   12. Known inherited or acquired bleeding and thrombophilia (e.g., hemophiliacs, coagulation dysfunction, thrombocytopenia, and hypersplenism);   13. Unhealed wound or fracture for a long time (pathologic fracture caused by a tumor is not considered);   14. Major surgical operation, severe traumatic injury, fracture, or ulceration within 4 weeks of enrollment;   15. Factors significantly affecting oral medication such as swallowing difficulty, chronic diarrhea, and intestinal obstruction;   16. An abdominal fistula, gastrointestinal perforation, or intraperitoneal abscess within 6 months before enrollment;   17. Urinary protein ≥++, and confirmed 24-hour urinary protein >1.0 g;   18. Serous effusion with clinical symptoms requiring symptomatic treatment (including hydrothorax, ascites, and hydropericardium);   Note: Asymptomatic patients with serous effusions could be enrolled; symptomatic patients with serous effusions who are treated with symptomatic treatment (no anticancer drugs used for serous effusion), and then could be enrolled as judged by the investigators;   - 1. Active infections require antimicrobial therapy (e.g., antibacterial drugs, antiviral drugs, excluding anti-hepatitis B therapy for chronic hepatitis B, and antifungal drug treatment);   2. Active hepatitis B (HBV DNA ≥2000IU/mL or 10^4^ copies/mL) or hepatitis C (hepatitis C antibody-positive, and HCV RNA above the lower limit of assay);   3. A history of psychotropic substance abuse with the inability to quit, or dysphrenia;   4. Participation in other clinical trials of antitumor drugs within 4 weeks prior to the study;   5. Patients with previous or co-existing uncured malignancies (other than skin basal cell carcinoma, cured cervical carcinoma in situ, and superficial bladder cancer);   6. Patients who had received a potent CYP3A4 inhibitor within 7 days prior to randomization, or had received a potent CYP3A4 inducer within 12 days prior to the study;   7. Pregnant or lactating women; A fertile patient who is unwilling or unable to use effective contraception;   8. Other conditions that may affect proceeding of the clinical study and its outcomes according to the investigator’s judgment. |
| Study Procedures | Group A (experimental group): 250 mg gefitinib, po, qd + 12 mg anlotinib hydrochloride capsule, po, qd, D1-D14; administered under fasting conditions (administration of the two drugs at the same time every day), every 3 weeks;  Group B (control group): 250 mg gefitinib, po, qd + anlotinib hydrochloride placebo, 1 tablet, po, qd, D1-D14; administration of the two drugs at the same time every day taking medication at the same time every day), every 3 weeks.  Drugs will be administered every 21 days until disease progression, intolerable toxicity, withdrawal of consent, or drug discontinuation according to the investigator's judgment. |
| Endpoints | Efficacy is evaluated every 2 cycles (6 weeks) after enrollment and every 3 cycles (9 weeks, 63±7 days) from 27 cycles using RECIST 1.1 criteria;  **Primary Endpoints:**  Progression-free survival (PFS)  **Secondary Endpoints:**  1. Overall Survival (OS)  2. Overall Response Rate (ORR)  3. Disease control rate (DCR)  4. Time to disease progression (TTPD)  5. Duration of Response (DoR)  6. Quality of life score (QoL) |
| Safety | Any adverse events (AEs) that occurred in all subjects during the clinical study period will be observed, including abnormal clinical symptoms and vital signs, and abnormalities in laboratory tests. The clinical characteristics, severity, time of occurrence, duration, treatment methods, and prognosis of adverse events will be recorded; meanwhile, the correlation between adverse events and the study drugs will be determined. The safety of drugs is evaluated according to National Cancer Institute's Common Terminology Criteria for Adverse Events (NCI-CTC AE) version 4.0. |
| Exploratory Study | The mechanism of drug resistance will be monitored by next-generation sequencing (NGS) before treatment, at the first efficacy evaluation, and during tumor progression. |
| Statistical Analysis | **Efficacy analysis**  For the PFS (primary endpoint), the median value and 95% confidence interval (CI) are estimated and survival curves are obtained using the Kaplan-Meier method. Survival differences between the two groups are estimated by log-rank tests. COX regression analysis is used to compare the survival between the two groups after adjusting for stratification factors such as EGFR mutation site, gender, PS score, and pathological type; the hazard ratio (HR) and 95% CI after adjusting for the above stratification factors are calculated.  For the secondary endpoints, such as OS, TTPD, and DoR, the median value and 95% CI are estimated and survival curves are obtained using the Kaplan-Meier method. The log-rank test is used to compare the survival between the two groups; DCR and ORR with 95% CI are calculated, and comparisons between treatment groups are performed using a Cochran-Mantel-Haenszel (CMH) test or chi-square test. Two independent samples will be compared for the differences between the two groups in QoL scores by the t-test or Wilcoxon rank sum test.  **Safety** **analysis**  Descriptive statistical analysis is mainly used, and the mean or incidence of examination results before and after administration could be compared if necessary. Vital signs, physical examination, and laboratory tests mainly describe the changes before and after treatment and the relationship between abnormal changes and study drugs. All AEs are standardized by MedDRA code, and the incidences of AEs and grade 3 or above AEs are summarized and compared between the two groups according to system organ class (SOC) and preferred language (PT) in MedDRA code and CTCAE classification. The AEs and serious AEs occurred in this study are also summarized in the table. |
| Prespecified Efficacy Analysis | An Independent Data Monitoring Committee (IDMC) will be established to conduct 1-2 interim analyses to provide further recommendations and to ensure the safety of subjects.  Final analysis: It is performed when approximately 192 PFS events have occurred |
| Study Schedule | Enrollment date of the first subject: January 2019  Enrollment date of the last subject estimated: June 2021  Estimated date of the end of the study: December 2022 |

**List of abbreviations**

| **Abbreviations** | **Full term** |
| --- | --- |
| ACE-I | Angiotensin-converting enzyme inhibitors |
| AE | Adverse event |
| AKP | Alkaline phosphatase |
| ALT | Alanine aminotransferase |
| ANC | Absolute neutrophil count |
| APTT | Activated partial thromboplastin time |
| AST | Aspartate aminotransferase |
| BIL | Bilirubin |
| BP | Blood pressure |
| BUN | Blood urea nitrogen |
| CFDA | China Food and Drug Administration |
| Cr | Creatinine |
| CR | Complete remission |
| CRF | Case Report Form |
| CT | Computed tomography |
| DBIL | Direct bilirubin |
| DCR | Disease control rate |
| DLT | dose-limiting dose |
| EC | Ethics Committee |
| ECOG PS | Eastern Cooperative Oncology Group performance status |
| EGFR | Epidermal growth factor receptor |
| FAS | Full analysis set |
| FIB | Fibrinogen |
| FISH | Fluorescence in situ hybridization |
| Glu | Glucose |
| GCP | Good Clinical Practice |
| GGT | Gamma-glutamyltransferase |
| h | Hour |
| Hb | Hemoglobin |
| HFS | Hand-foot syndrome |
| HR | Heart rate |
| IB | Investigator's brochure |
| IBIL | Indirect bilirubin |
| INR | International normalized ratio |
| ITT | Intention-to-treat |
| IU | International unit |
| IV | Intravenous injection |
| LDH | lactate dehydrogenase |
| MRI | Magnetic resonance imaging |
| MTD | Maximum tolerated dose |
| MVD | Microvessel density |
| NCI-CTC | National Cancer Institute Common Toxicity Criteria |
| OB | Occult blood |
| ORR | Overall response rate |
| OS | Overall survival |
| PD | Progressive disease |
| PFS | Progression-free survival |
| PLT | Platelet |
| PR | Partial response |
| PT | Prothrombin time |
| qd | Once a day |
| RBC | Red blood count |
| RECIST | Response Evaluation Criteria In Solid Tumors |
| RR | Response rate |
| RTKs | Receptor tyrosine kinases |
| SAE | Serious adverse event |
| SAP | Statistical analysis plan |
| SAS | Safety analysis set |
| SBP | Systolic blood pressure |
| SD | Stable disease |
| SVR | Vascular resistance |
| TBIL | Total bilirubin |
| TP | Total protein |
| TT | Thrombin time |
| UNL | Upper normal limit |
| VEGF | Vascular endothelial growth factor |
| VEGFR | Vascular endothelial growth factor receptor |
| WBC | White blood cell count |

1. Background
   1. **Overview of lung cancer and targeted therapy**

Lung cancer currently has the highest morbidity and mortality among all malignant tumors, of which non-small cell lung cancer (NSCLC) accounts for about 80%-85% of lung cancer, and about 30%-40% of NSCLC patients have advanced disease at diagnosis [[1](#_ENREF_1), [2](#_ENREF_2)]. For patients with advanced NSCLC, standard two-drug platinum-containing chemotherapy has reached a plateau of efficacy, and the median overall survival (OS) of patients was only 10 months [[3](#_ENREF_3)]. In recent years, targeted therapy drugs -- epidermal growth factor receptor tyrosine kinase inhibitors (EGFR-TKIs) showed a strong inhibitory effect on tumor growth and few side effects. The development and application of these drugs have triggered an epoch-making revolution in the treatment of advanced lung cancer, which has become a hot spot and brought new hope to patients with advanced lung cancer. These drugs have significantly prolonged the progression-free survival (PFS), OS, and quality of life in advanced patients, which have demonstrated clinical benefits for patients with EGFR mutations. The median OS reached 24 months in these patients after treatment, and the treatment was well-tolerated and led to significant improvements in quality of life.

The first-generation EGFR-TKIs include gefitinib, erlotinib, and icotinib. Among them, gefitinib is the first TKI proven as monotherapy for EGFR mutation-positive advanced NSCLC. Large international multi-center phase III clinical trials (IPASS, First-SIGNAL, WJTOG3405, and NEJ002) [[4-7](#_ENREF_4)] have confirmed that gefitinib can significantly prolong the PFS compared with standard chemotherapy in this patients population, which is recommended by multiple guidelines worldwide. Since gefitinib marketed in China for 10 years, studies have confirmed that the median OS of Chinese patients with advanced lung adenocarcinoma increased from 14.1 months to 33.5 months after its approval. As a first-line treatment for EGFR-mutated advanced NSCLC in Asian and Caucasian populations, gefitinib has been effective and well-tolerated (median PFS, 8.4-10.8 months; overall response rate [ORR], 62.1-84.6%; median OS, 21.6-34.8 months) [[4-7](#_ENREF_4)]. Therefore, all NCCN guidelines for NSCLC since 2011 recommend EGFR-TkIs as the first-line treatment in patients with EGFR-mutated advanced NSCLC [[8](#_ENREF_8)].

Although these drugs are currently the most mature molecular targeted drugs for the treatment of advanced NSCLC, the most troublesome problem is that most of the patients who responded to the initial treatment had tumor progression after a median response of 10 months after receiving first-generation EGFR-TKIs [[9](#_ENREF_9), [10](#_ENREF_10)]. After the failure of first-line treatment, the third-generation EGFR-TKIs (Tagrisso, AZD9291) can be used for patients with T790M mutation resistance according to the resistance mechanism. Chemotherapy remains the standard treatment for patients with non-T790M mutation after drug resistance. In addition, osimertinib as the first-line treatment could also significantly delay the time of drug resistance, with a PFS of 18.9 months. Therefore, the third-generation EGFR-TKI osimertinib has been approved for second-line treatment and first-line treatment after TKI resistance. Many patients choose to give up treatment after the failure of first-line targeted therapy, which brings serious challenges to the improvement of overall survival. Even patients who can choose the third-generation targeted drugs will bear a heavy economic burden, and many patients and their families will become poor due to the disease.

According to the current treatment mode, the use of EGFR-TKIs alone in the treatment of advanced NSCLC has reached the therapeutic platform. The resistance to targeted drugs has become a key factor limiting the survival benefit of patients and bringing serious psychological and economic burdens to patients. Therefore, finding strategies to delay and overcome drug resistance has become a key hot issue in the field of lung cancer.

In recent years, new therapeutic strategies of anti-tumor drugs emerge in an endless supply, which not only target tumor cells but also the tumor microenvironment, especially tumor angiogenesis killing tumors in all directions. Anti-angiogenic therapy specifically binds to vascular endothelial growth factor (VEGF) and prevents it from interacting with its receptors, thus exerting a variety of effects on tumor blood vessels. This class of drugs includes monoclonal antibodies and small-molecule targeted drugs. In the first-line treatment of advanced NSCLC, ECOG4599 [[11](#_ENREF_11)], a large phase III randomized controlled trial reported in 2006, showed that the median PFS/OS of bevacizumab plus paclitaxel (200 mg/m^2^) and carboplatin were 2-2.5 months longer than that of chemotherapy alone; one-year survival improved by 10%. Based on the positive results of this study, the bevacizumab/paclitaxel/carboplatin regimen has been established as the new standard regimen of ECOG treatment for advanced NSCLC. Bevacizumab combined with chemotherapy is recommended for the first-line treatment of NSCLC. In October 2006, the US Food and Drug Administration (FDA) approved the drug plus paclitaxel/carboplatin regimen as the first-line treatment of advanced non-squamous NSCLC. The BEYOND [[12](#_ENREF_12)] study published in 2013 first confirmed that bevacizumab combined with carboplatin plus paclitaxel was an effective and safe treatment regimen for Chinese patients with non-squamous NSCLC. This study confirmed a median PFS improvement of 2.7 months with bevacizumab plus chemotherapy compared with chemotherapy alone. In July 2015, the China Food and Drug Administration (CFDA) approved bevacizumab in combination with paclitaxel/carboplatin regimen for the first-line treatment of advanced non-squamous NSCLC.

In addition to the survival benefit of bevacizumab combined with chemotherapy, the results of the Japanese study JO25567 [[13](#_ENREF_13)] reported in 2014 compared the efficacy of erlotinib plus bevacizumab and erlotinib monotherapy in the first-line treatment in patients with EGFR mutation-positive advanced NSCLC. A total of 152 patients with EGFR mutation-positive NSCLC who received initial treatment were randomly assigned to receive erlotinib alone (n=77) or erlotinib combined with bevacizumab (n=75). Results showed that the median PFS was 9.7 months (95% CI 5.7-11.1) in the erlotinib monotherapy group and 16.0 months (95% CI 13.9-18.1) in the erlotinib combined bevacizumab group, and PFS improved by 6.3 months in the combination group (*P*=0.0015; HR=0.54) [[13](#_ENREF_13)]. In terms of adverse events (AEs), grade 3 or 4 rash was more common in patients in the erlotinib plus bevacizumab group (25.3% in the combination group vs. 19.5% in the monotherapy group), and grade 3 or 4 bleeding was more common but less common (2.7% in the combination group vs. 0.0% in the monotherapy group). Overall, toxicity was tolerable with a significant survival benefit in the combination group. Recently, the 2018 American Society of Clinical Oncology (ASCO) Annual Meeting reported the efficacy and safety of erlotinib plus bevacizumab compared with erlotinib monotherapy as the first-line treatment for EGFR mutation-positive advanced NSCLC. Erlotinib plus bevacizumab had significantly longer PFS than erlotinib monotherapy (16.9 vs 13.3 months, *P*<0.01), which suggested that EGFR-TKIs combined with anti-angiogenesis therapy could bring longer PFS to patients and delay the occurrence of drug resistance to targeted therapy.

However, bevacizumab requires intravenous administration every three weeks and it is not convenient for patients and may cause hypersensitivity reactions, which affects clinical promotion to a certain extent. In recent years, small-molecule anti-angiogenic drugs have become a new hotspot in the field of targeted therapy. Apatinib (YN968D1), a potent VEGFR2 tyrosine kinase inhibitor, can block VEGF-induced endothelial cell migration and proliferation and reduce tumor microvessel density. It mainly plays an antiangiogenic role in the treatment of malignant tumors by inhibiting VEGFR2. In 2014, apatinib mesylate tablets were approved by State Food and Drug Administration (SFDA) for third-line treatment of advanced gastric cancer [[14](#_ENREF_14)]. We just completed a phase I study of apatinib plus gefitinib as first-line treatment for EGFR-mutation-positive NSCLC and found that anlotinib plus gefitinib did significantly prolong PFS in patients with 19 months in 6 patients in the 500 mg dose group and 13.4 months in 6 patients in the 250mg dose group. In terms of safety, grade 3 or above AEs occurred in 4 patients (33%), and among them were 2 patients with 3 degrees of transaminase, which recovered after discontinued administration to protect the liver with no dose reduction. One patient had urinary occultic blood (+++), and one patient was excluded from the study due to hypertension (drug-related serious adverse events [SAE]) with an incidence of 8% (1/12) and has been withdrawn from the group at present. Overall, the treatments were well-tolerated with a manageable safety profile. Based on the results of the preliminary study, we are conducting a phase III randomized, controlled, double-blind study comparing apatinib plus gefitinib versus placebo plus gefitinib. More than 240 patients have been enrolled and study enrollment is coming to an end.

Anlotinib (AL3818) hydrochloride is a highly potent multi-target tyrosine kinase inhibitor of VEGFRs, FGFRs, and PDGFRs, which can effectively block the migration and proliferation of endothelial cells and reduce the density of tumor microvessels. This drug exerts antiangiogenic effects mainly by inhibiting VEGFRs, FGFRs, and PDGFRs, and achieves direct antitumor effects indirectly and against potential targets. Anlotinib, a novel drug for targeted therapy independently developed in China, is the first small-molecule targeted anti-angiogenic drug proved to be safe and effective as monotherapy in advanced lung cancer worldwide. The drug can significantly prolong the survival of patients with advanced lung cancer after treatment failure on standard second-line chemotherapy regimens. Anlotinib has been approved by the CFDA as a third-line treatment for advanced NSCLC [[15](#_ENREF_15)].

Compared with apatinib, anlotinib can simultaneously inhibit angiogenic targets including VEGFRs, FGFRs, and PDGFRs, while apatinib mainly acts on VEGFR2; thus, anlotinib shows stronger antiangiogenic activity than apatinib. The reported efficacy data (ALTER-0303 study) showed a significantly prolonged median OS (9.63 vs. 6.3, HR=0.68, *P*<0.0018) and median PFS (5.37 vs. 1.40, HR=0.25, *P*<0.0001), as well as a significantly improved ORR (9.18% vs. 0.7%) and disease control rate (DCR; 81% vs. 37%) in patients with NSCLC treated with anlotinib as a second-line treatment compared to placebo. However, apatinib similarly as a second-line treatment of NSCLC was less effective, with a PFS of 4.7 months, a response rate of 12%, and a DCR of 61%. In addition to being less effective than anlotinib, anlotinib is also reported to be safer than apatinib. A phase III study reported a 13.2% incidence of grade 3-5 hypertension, 3.7% of dermal toxicity, and 2.4% of albuminuria, while that hypertension, albuminuria, and hand-foot syndrome caused by apatinib were found to be more serious in our previous phase II study of lung cancer.

Based on the above evidence, patients with EGFR-mutation-positive advanced NSCLC will be enrolled in this study. Treat-naive patients will be randomly treated with gefitinib plus anlotinib or gefitinib plus placebo in a 1:1 ratio. The efficacy and toxicity are regularly evaluated, and treatment is continued until unacceptable toxicity and disease progression. Efficacy, survival, and safety are compared between the two groups. This study aims to provide high-level evidence-based medical evidence for the advantages of EGFR-TKIs combined with anti-angiogenic targeted drugs, providing a new regimen for further improving the efficacy of first-line treatment in patients with EGFR-mutation-positive advanced NSCLC (Chia Tai Tianqing Pharmaceutical Group Co., Ltd has agreed to provide free anlotinib and placebo for this study. Patients have the opportunity to receive free anlotinib treatment, which may bring significant survival benefits).

- 1. **Drug information**

**Overview of drug**

Common name: Anlotinib Hydrochloride Capsules

English name: Anlotinib Hydrochloride Capsules

Bopomofo: Yansuan Anluotini Jiaonang

Anlotinib hydrochloride is a hard capsule. The chemical structure of anlotinib is as follows:

Molecular formula: C_23_H_22_FN_3_O_3_·2HCl Molecular weight: 480.36

**Figure. 1** **Structural formula for anlotinib**

Anlotinib hydrochloride is a multi-target receptor tyrosine kinase inhibitor. 1) It has obvious inhibitory activity against vascular formation-related kinases such as VEGFR1/2/3, FGFR1/2/3, and other tumor-related kinases related to cell proliferation such as PDGFRα/β, c-Kit, and Ret. Inhibition of angiogenic kinase has a broader spectrum (e.g., Met, FGFR1/2/3). 2) It also has obvious inhibitory activity against some kinase targets such as Aurora-B, c-FMS, and DDR1 in the ongoing study. 3) It has obvious inhibitory activity on a variety of kinase mutants such as PDGFRα, cKit, Met, and EGFR, and it shows even stronger inhibitory activity on the mutant than that on the wild-type.

- 1. **Pharmacological study of anlotinib**

No significant effect on the general behavior and spontaneous activity of mice gavaged with a single dose of 2, 6, and 20 mg/kg after 24 hours was observed. The Beagle dogs were given different concentrations of anlotinib hydrochloride (0.3, 0.9, 3 mg/kg) through the duodenum after anesthesia. The abnormal systolic blood pressure (BP), diastolic blood pressure, mean arterial pressure, and arrhythmia of anesthesia dogs were not observed in each dose group within 240 minutes after dosing. In parallel, anlotinib had no obvious effects on heart rate, PR, QRs, QTc interval of electrocardiograms (ECG), respiratory rate, and respiratory amplitude.

- 1. **Toxicology of anlotinib**

**1.4.1 Acute toxicity test**

After ICR mice were gavaged with a single dose of anlotinib, the LD50 at days 1-14 was 1735.9 mg/kg with a 95% confidence limit of 1365.5-5474.6 mg/kg, and the LD50 at days 1-22 was 982.8 mg/kg with a 95% confidence limit of 657.28-1180.3 mg/kg. Possible target organs were the liver, gallbladder, small intestine (mainly duodenum), kidney, spleen, and testis. The Beagle dogs were given anlotinib as single intragastric administration and were then suspended and observed for 14 days. The MTD was 20 mg/kg and the minimum lethal dose was 67.5 mg/kg. The drug-related toxicities resembled those reported in similar drugs.

**1.4.2 Long-term toxicity test**

The SD rats were given anlotinib orally for 13 weeks and suspended for 6 weeks. The NOAEL was 0.25 mg/kg and the toxic dose was ≥1 mg/kg. The target organs were teeth, hepatobiliary system, duodenum, pancreas, adrenal gland, kidney, and blood system. The lesions of the hepatobiliary system, teeth, and blood system recovered significantly after discontinuance, while the lesions of other organs recovered completely.

The Beagle dogs were given anlotinib by intragastric administration for 13 weeks and suspended for 4 weeks. The main toxicities at the dose of 0.40 mg/kg (AUC_0→8h_ 195 ng^.^h/mL) were gastrointestinal reactions, slight slowing of heart rate, and influence on liver and kidney functional indexes. The NOAEL was 0.12 mg/kg with the AUC_0→8h_ of 51.7 ng^.^h/mL. After discontinuance for 4 weeks, all the above toxicities were recovered, and no delayed toxic reactions were observed.

**1.4.3 Mutagenicity test and previous literature**

Anlotinib had no mutagenic effect on Salmonella typhimurium in Salmonella Typhimurium Histidine Reversion Test (Ames Test). Anlotinib did not induce chromosome structural aberration of Chinese hamster lung fibroblast cells in the chromosome aberration test. Anlotinib did not induce the increased micronucleus rate of mice marrow polychromatic erythrocytes in the micronucleus test.

**1.4.4 Reproductive toxicity test and previous literature**

The NOAEL for fetal development was <0.3 mg/kg when pregnant SD rats were intragastrically administered with anlotinib.

- 1. **Pharmacokinetics of anlotinib**

**1.5.1 Overview of preclinical pharmacokinetics in animals**

**Pharmacokinetic parameters:**

Results of plasma pharmacokinetic studies in rats and dogs indicated slower absorption of anlotinib hydrochloride in the gastrointestinal tract after oral administration. Bioavailability was ~34% in rats and 67% in dogs.

**Plasma protein binding rate:**

The binding rates between anlotinib and plasma protein in rats, dogs, and humans were 97%, 96%, and 93%, respectively. The rates did not change with the drug concentration of anlotinib.

**Tissue distribution:**

The drug concentration in all tissues was higher than that in blood at the same time, and there was no significant difference in the tissue distribution of anlotinib. The peak concentration in the lung was 184 (male) and 331 (female) times the plasma concentration at the same time. The peak concentrations in the spleen, adrenal gland, large intestine, small intestine, ovary, and kidney were 65-144 times the plasma concentration. The peak concentrations in the uterus, heart, liver, stomach, bladder, bone marrow, and fat were 20-47 times the plasma concentration. The peak concentrations in skeletal muscle, pancreas, testis, and brain were 1.7-13 times plasma concentration.

**Tissue distribution in tumor-bearing mice:**

After 4 hours of oral administration, the concentration of anlotinib in the tissue of tumor-bearing nude mice was the highest. The AUC of anlotinib in all tissues was positively correlated with the dose, and the AUC in liver tissue was linearly correlated with the dose. The concentrations of anlotinib in the lung and liver were the highest (~10-14 times plasma AUC), followed by the kidney (~5.9-8.6 times plasma AUC). The AUC of anlotinib in the tumor was ~2.4-2.6 times of plasma AUC. The concentration in the colon was similar to that in plasma (~0.8-1.0 times plasma AUC).

**Excretion:**

The cumulative amount of anlotinib excreted (Cum. Ae) by urine (0-72 h), feces (0-72 h), and bile (0-24 h) was less than 5% of the injection dose (1.5 mg/kg). It suggested that metabolic transformation was the main pathway of elimination of anlotinib.

**Metabolite:**

Twenty-three metabolites were detected in rat bile; 16 metabolites were detected in rat urine; 12 metabolites were detected in feces; 8 metabolites were detected in plasma. Thereinto, M16, M21, and M23 were the 3 primary plasma metabolites. The cumulative excretion of M16 was the highest in urine and bile, while that of M18 was the highest in feces.

**Metabolic enzyme activity:**

Anlotinib could reversibly inhibit 7 CYP enzymes (8 substrates) in human liver microsomes, with IC50 values of >100 μM (CYP1A2), 4.96 μM (CYP2B6), 3.71 μM (CYP2C8), 1.56 μM (CYP2C9), 1.67 μM (CYP2C19), 24.7 and 18.5 μM (CYP2D6), 9.56 μM (CYP3A4-midazolam), and 2.09 μM (CYP3A4-testosterone), respectively.

**1.5.2 Phase I study of pharmacokinetics**

The phase I tolerability and preliminary efficacy study enrolled subjects with malignant solid tumors who had been diagnosed and failed or lacked standard therapy to observe the tolerability of anlotinib hydrochloride capsule.

**1.5.2.1 Tolerability of anlotinib with consecutive 2 weeks and stop 1 week**

Preliminary studies found that anlotinib showed a long elimination half-life, which the drug was accumulated in subjects under continuous administration, and many AEs occurred when 10 mg was used for a cycle of 28 consecutive days. Considering the tolerability of the subjects, the administration regimen with consecutive 2 weeks and stop 1 week (every 3 weeks [21 days], at least 2 cycles [42 days]) was explored for tolerance observation. The results of the study are as follows：

- **10 mg qd of anlotinib with consecutive 2 weeks and stop 1 week：**

A total of 3 subjects were observed for administration in this study protocol, and AEs occurred within 2 cycles were as follows:

**Table 1. Adverse events of anlotinib (10 mg) in 2 cycles of administration regimen**

| **Adverse events (AEs)** | **N＝3** | **Criteria** |
| --- | --- | --- |
| At least one AE occurs | 3 | － |
| Increased fat and amylase | 1 | Grade III |
| Fatigue | 1 | Grade II |
| Increased blood pressure | 1 | Grade I |
| Diarrhea and abdominal pain | 1 | Grade I |
| Hoarseness | 2 | Grade I |

In the one subject with abnormal amylase mentioned above, abnormally elevated lipase and amylase occurred after 5 cycles of administration, which fluctuated in the range of grade I-III, with the highest level of grade IV lipase and grade III amylase. During the study, subjects were treated with anti-inflammatory tablets until tumor progression (118 weeks).

During the administration after 2 cycles, grade II AEs included 1 diarrhea and leukopenia, and the other grade I AEs included 1 headache/dizziness, 1 hand-foot skin reaction, 1 leukopenia, proteinuria, and elevated aminotransferase.

- **16 mg qd of anlotinib with consecutive 2 weeks and stop 1 week:**

A total of 3 subjects were enrolled in this administration regimen, and 1 subject experienced grade III hypertension (week 3) and grade III fatigue within 2 cycles of treatment. The investigators considered 16 mg to be the dose-limiting dose (DLT) of anlotinib in this administration regimen. Other AEs occurring within 2 cycles are listed in the table below.

**Table 2. Adverse events of anlotinib (16 mg) in 2 cycles of administration regimen**

| **Adverse events (AEs)** | **N＝3** | **Criteria** |
| --- | --- | --- |
| At least one AE occurs | 3 | － |
| Fatigue (DLT) | 1 | Grade III |
| Increased blood pressure (DLT) | 1 | Grade III |
|  | 1 | Grade II |
| Hypothyroidism | 2 | Grade II |
| Elevated ALT and AST | 1 | Grade II |
| Bleeding | 1 | Grade II |
| Diarrhea and abdominal pain | 1 | Grade I |
| Elevated triglyceride | 2 | Grade I |
| Hoarseness | 1 | Grade I |

After 2 cycles of administration, the following AEs occurred in 1 subject, including grade II bleeding (the dose was subsequently reduced to 12 mg for further observation), elevated bilirubin, hypertension, elevated transaminase, and hypothyroidism. Grade I AEs included proteinuria, hoarseness, diarrhea, toothache, and tinnitus.

- **12 mg qd of anlotinib with consecutive 2 weeks and stop 1 week:**

Considering the large range of 10 and 16 mg qd, the investigators further explored the maximum tolerated dose of 12 mg qd. After the tolerance observation of 21 subjects successively, the 12 mg qd regimen with consecutive 2 weeks and stop 1 week was recognized as the recommended regimen for subsequent studies, and the 12 mg qd regimen was the maximum tolerated dose.

The possible drug-related AEs during the study (as of July 1, 2014, 8 subjects were still on medication) were counted according to the first 2 cycles and the whole process of treatment, as detailed in the following table. During administration in this dose group, a total of 5 subjects had 7 times of grade III AEs: elevated triglyceride in subject 24, hypertension and hand-foot skin reaction in subject 29, increased lipase in subject 31, elevated bilirubin in subject 32, and hypertension and elevated triglyceride in subject 34.

**Table 3. Adverse events of anlotinib (12 mg) in 2 cycles of administration regimen**

| **Adverse events (AEs)**  **N=21 n (%)** | **Grade I/II** | | **Grade III** | |
| --- | --- | --- | --- | --- |
|  | **First 2 cycles** | **The whole study process** | **First 2 cycles** | **The whole study process** |
| At least one AE occurs | 21 (100%) | 21 (100%) | 2 (9.52%) | 7 (33.33%) |
| Hand-foot skin reaction | 4 (19.05%) | 10 (47.62%) | 0 | 1 (4.76%) |
| Rash | 4 (19.05%) | 6 (28.57%) | 0 | 0 |
| Increased blood pressure | 5 (23.81%) | 5 (23.81%) | 0 | 2 (9.52%) |
| Albuminuria | 5 (23.81%) | 14 (67%) | 0 | 0 |
| Elevated triglyceride | 6 (28.57%) | 11 (52.38%) | 1 (4.76%) | 2 (9.52%) |
| Elevated total cholesterol | 6 (28.57%) | 13 (62%) | 0 | 0 |
| Elevated low-density lipoproteins | 4 (19.05%) | 11 (52.38%) | 0 | 0 |
| Hypothyroidism | 8 (38.10%) | 12 (57 %) | 0 | 0 |
| Hyperthyroidism | 2 (9.52%) | 2 (9.52%) | 0 | 0 |
| Elevated thyrotropin | 2 (9.52%) | 4 (19.05%) | 0 | 0 |
| Elevated ALT | 6 (28.57%) | 10 (47.62%) | 0 | 0 |
| Elevated AST | 4 (19.05%) | 9 (42.86%) | 0 | 0 |
| Elevated creatinine | 1 (4.76%) | 2 (9.52%) | 0 | 0 |
| Elevated total bilirubin | 5 (23.81%) | 8 (38.10%) | 0 | 0 |
| Increased direct bilirubin | 3 (14.29%) | 8 (38.10%) | 0 | 1 (4.76%) |
| Increased indirect bilirubin | 4 (19.05%) | 5 (23.81%) | 0 | 0 |
| Lipase | 1 (4.76%) | 5 (23.81%) | 1 (4.76%) | 1 (4.76%) |
| Blood amylase | 4 (19.05%) | 9 (42.86%) | 0 | 0 |
| Abnormal myocardial enzyme | 2 (9.52%) | 3 (14.29%) | 0 | 0 |
| Leukopenia | 3 (14.29%) | 6 (28.57%) | 0 | 0 |
| Neutropenia | 0 | 2 (9.52%) | 0 | 0 |
| Thrombocytopenia | 0 | 2 (9.52%) | 0 | 0 |
| Bleeding | 0 | 1 (4.76%) | 0 | 0 |
| Urine occult blood | 5 (23.81%) | 8 (38.10%) | 0 | 0 |
| Fatigue | 5 (23.81%) | 7 (33.33%) | 0 | 0 |
| Diarrhoea | 6 (28.57%) | 7 (33.33%) | 0 | 0 |
| Hoarseness | 3 (14.29%) | 5 (23.81%) | 0 | 0 |
| Nausea | 3 (14.29%） | 3 (14.29%) | 0 | 0 |
| Anorexia | 1 (4.76%) | 2 (9.52%) | 0 | 0 |
| Toothache | 1 (4.76%) | 4 (19.05%) | 0 | 0 |
| Gingivitis | 1 (4.76%) | 1 (4.76%) | 0 | 0 |
| Pain | 4 (19.05%) | 4 (19.05%) | 0 | 0 |
| Throat pain | 1 (4.76%) | 4 (19.05%) | 0 | 0 |
| Dizziness/headache | 1 (4.76%) | 2 (9.52%) | 0 | 0 |
| Fever | 1 (4.76%) | 2 (9.52%) | 0 | 0 |
| Oral mucositis | 0 | 2 (9.52%) | 0 | 0 |
| Tinnitus | 1 (4.76%) | 1 (4.76%) | 0 | 0 |
| Premature beat | 0 | 1 (4.76%) | 0 | 0 |

**1.5.2.2 Pharmacokinetics in humans**

According to the protocol of the phase I study, all solid tumor subjects participating in the early tolerance study received blood drug concentration monitoring using liquid-mass combination technology (LC/MS/MS). The results were used to estimate the pharmacokinetic parameters of anlotinib hydrochloride.

- **Pharmacokinetics of a single dose**

Eligible subjects were given anlotinib once for a single-dose clinical pharmacokinetic study. Plasma concentrations of 19 subjects in 3 dose groups (10 mg, 16 mg, and 12 mg) were detected. The mean time curve of each dose group was shown on the left of **Figure 1.5-1**. The pharmacokinetic parameters estimated were shown in Table.

After single oral administration of anlotinib, anlotinib reached a high level of plasma concentration in 4-8 h and achieved a long half-life. At the dose of 10, 12, and 16 mg/person, the in vivo exposure of anlotinib (AUC_0-168h_) was positively correlated with the dose, but the linear correlation was uncertain.

After a single oral administration, cumulative urinary excretion of anlotinib was ~<4% of the oral dose.

**Table 4. Pharmacokinetics of single administration of anlotinib in humans**

| **Pharmacokinetic parameters** | **Mean ± SD (RSD %)** | | |
| --- | --- | --- | --- |
|  | **10 mg/person (n=4)** | **12 mg/person (n=11)** | **16 mg/person (n=4)** |
| C_max_ (ng/mL) | 5.78 ± 2.76 (47.7) | 10.5 ± 2.9 (28.0) | 15.8 ± 3.2 (20.1) |
| T_max_ (h; po) | 6.0 ± 4.4 (73.3) | 7.3 ± 3.3 (45.6) | 11.0 ± 8.9 (80.6) |
| AUC_0-t_ (ng∙h/mL) | 385 ± 175 (45.7) | 875 ± 240 (27.5) | 1290 ± 384 (29.7) |
| AUC_0-∞_ (ng∙h/mL) | 562 ± 328 (58.3) | 1066 ± 263 (24.6) | 1585 ± 470 (29.6) |
| t_1/2_ (h) | 95.3 ± 21.7 (23) | 116 ± 47 (40.5) | 97.9 ± 14.8 (15.1) |

- **Pharmacokinetics of multiple doses**

Eligible subjects received anlotinib once for a single-dose clinical pharmacokinetic study. Thereafter, the pharmacokinetic study with continuous administration was initiated following a ≥7-day wash-out period. Subjects received anlotinib on days 1-14 every 3 weeks and were monitored for at least 2 cycles. A total of 21 subjects in 3 dose groups (10 mg, 16 mg, and 12 mg) were given anlotinib and monitored the plasma concentration meanwhile. The mean time curve of each dose group was shown on the right of **Figure 1.5-1**. The pharmacokinetic parameters estimated were shown in Table.

After multiple doses, the plasma concentration of anlotinib increased along with the administration times due to the long elimination half-life of anlotinib in humans. The administration regimen with consecutive 2 weeks and stop 1 week was applied to control the plasma concentration of anlotinib following the administration. Anlotinib reached its maximum plasma concentration on day 14 following the administration regimen. The plasma concentration of anlotinib was almost <100 ng/mL at the dose of 10 and 12 mg/person/day.

**Table 5. Pharmacokinetics of anlotinib in 2 cycles of administration regimen**

| **Pharmacokinetic parameters** | **Mean ± SD (RSD %)** | | |
| --- | --- | --- | --- |
|  | **10 mg/person/day (n=3)** | **12 mg/person/day (n=15)** | **16 mg/person/day (n=3)** |
| C_max_ (ng/mL) | 65.2 ± 28.9 (44.3) | 61.6 ± 16.3 (27) | 93.7 ± 27.8 (30.0) |
| AUC_0-42_ (ng∙h/mL) | 1550 ± 864 (58) | 1467 ± 395 (27) | 2237 ± 814 (36.4) |


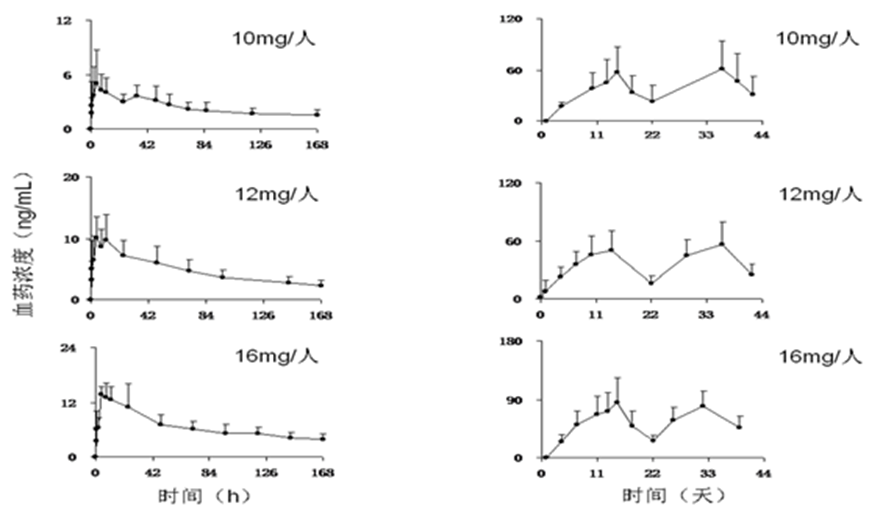


The time curve of a single dose The time curve of the administration regimen

**Figure 2. The time curve of a single dose or administration regimen**

- **Effect of food on the pharmacokinetics in healthy subjects**

The study was conducted through a randomized, two-period, self-crossover design. Twelve healthy subjects aged 18-40 were randomly and evenly divided into two groups: a fasting group with 3 males and 3 females and a high-fat diet group (3 males and 3 females). After fasting for 10 h, subjects were given 5 mg of anlotinib on an empty stomach or after a meal. Following a 28-day washout, subjects in the two groups were switched and the dose remained unchanged.

The pharmacokinetic parameters of administration in the fasting and postprandial state were similar, as detailed in Figure and Table. The results showed that, compared with fasting administration, the peak time of anlotinib in humans was extended following administration in the postprandial state, while the drug absorption was slightly reduced (~80% of fasting administration). Thus, it is recommended to take anlotinib in the fasting state in the subsequent clinical practice.


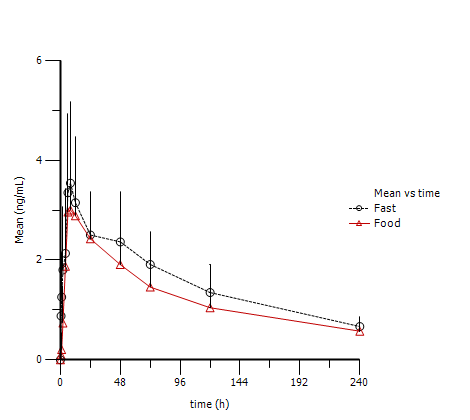


**Figure 3. Effect of food on the pharmacokinetics in healthy subjects**

**Table 6. Effect of food on the pharmacokinetic parameters in healthy subjects**

| **Pharmacokinetic parameters** | **High-fat diet group** | **Fasting group** |
| --- | --- | --- |
| C_max_ (ng·mL^-1^) | 3.35 ± 1.35 | 3.90 ± 1.60 |
| T_max_ (h) | 10.5 ± 6.7 | 9.3 ± 5.1 |
| AUC_last_/AUC_0-t_ (ng·h·mL^-1^) | 308 ± 120 | 377 ± 134 |
| AUC_inf_/AUC_0-∞_ (ng·h·mL^-1^) | 392 ± 145 | 486 ± 158 |
| MRT_last_/MRT_0-t_ (h) | 83.7 ± 6.8 | 86.0 ± 6.5 |
| MRT_inf_/MRT_0-∞_ (h) | 153.2 ± 43.7 | 161.7 ± 41.3 |
| t_1/2_ (h) | 107.2 ± 27.4 | 113.2 ± 30.3 |

- 1. **Clinical study**

As of April 2018, phase II and III clinical studies of anlotinib have been conducted in multiple indications, including medullary thyroid cancer, differentiated thyroid cancer, soft tissue sarcoma, NSCLC, small cell lung cancer, renal cancer, neuroendocrine tumor, colorectal cancer, liver cancer, gastric cancer, and phase I indication exploration. For detailed information on relevant clinical studies, please refer to the Investigator's Manual.

1. Study Objective

To observe and evaluate the efficacy and safety of anlotinib combined with gefitinib in patients with stage IIIB-IV EGFR-mutated NSCLC.

1. Study Design
   1. **Study design**

This study is a multicenter, randomized, double-blind, placebo-controlled, parallel clinical trial to evaluate the efficacy and safety of gefitinib combined with anlotinib as first-line treatment in patients with stage IIIB-IV EGFR-mutated NSCLC.

In the absence of withdrawal of consent, intolerable side effects, or a decision by the investigator to be inappropriate for further trial, treatment is continued for each subject until radiographically confirmed disease progression. The efficacy and safety indices are observed during the study.

- 1. **Type of comparison**

This study is a parallel, placebo-controlled study, and its type of comparison is a superiority trial.

PFS is used as a primary endpoint in this study. If the HR of the PFS comparison between the two groups is statistically significant and clinically significant, the study drug is considered superior to the placebo.

- 1. **Estimating sample size**

The primary outcome of this study was PFS after first-line therapy for lung cancer. Referring to the relevant clinical trial data and literature [[4-7](#_ENREF_4), [15](#_ENREF_15)], the median PFS of the control group (gefitinib + placebo) was 10 months, and the expected median PFS of the experimental group (gefitinib + anlotinib) was 15 months (the difference in PFS of 5 months could be considered clinically significant). Since the superiority test is used in this study, it is considered that the efficacy of the experimental group is better than that of the control group. If α=0.05 (two-sided) and 1-β=0.80 were set, the median PFS of the experimental group and the control group were 15 months and 10 months, respectively, and the ratio of patients in the two groups was 1:1. Assuming that the enrollment of subjects was completed within 18 months with 18-month follow-up period, two interim analyses are planned. Group-Sequential Log-rank Tests (Simulation) method using PASS 15 software showed that a total of 248 subjects (at least 124 subjects in each group) need to be enrolled, and a total of 192 PFS events were required to detect in the two groups (88 in the experimental group and 104 in the control group). If a dropout rate of 20% is considered, at least 155 subjects should be enrolled in each group, for a total of 310 subjects.

Patients with stage IIIB-IV EGFR-mutated NSCLC

Sign informed consent

筛选检查入组

**Random Balance**

EGFR mutation site, sex, PS score, and pathological type

**Experimental Group (A)**

Gefitinib + anlotinib (every 21 days)

**Control group (B)**

Gefitinib + placebo (every 21 days)

After the end of treatment, NGS is used to monitor the mechanism of drug resistance, and survival follow-up is continued.

The original regimen is continued until disease progression or intolerable adverse events.

Progressive disease

Shrinking or stable tumor

The efficacy will be assessed every 2 cycles (42 days) after initial administration and every 3 cycles (63 days) after cycle 27. Resistance mechanisms are monitored by NGS.

**Figure 3.7 Study design**

1. Randomization and Blinding
   1. **Randomization design**

The Interactive Web Response System (IWRS) is used for randomization in this study, and random numbers are automatically generated by statisticians using computer software. Subjects will be randomly assigned to the experimental and control groups in a 1:1 ratio. Stratification factors (in descending order of importance) included EGFR mutation sites (exon 19, 21), sex (male, female) and PS score (0, 1), pathological type (pure adenocarcinoma, and others).

The investigator is required to interact with the central randomization system after verifying that the participant meets the inclusion/randomization criteria and confirming the randomization stratification factors. The automated system will assign subjects to different groups according to applicable stratification criteria, and the investigator will immediately receive a response number from the central randomization system with a number assigned to the study drug package; meanwhile, an applicable study drug package is selected from the drug storage at the study center and distributed to subjects.

Neither the investigators nor anyone else within the study center is blinded to the study drugs administrated to subjects.

- 1. **Blinding design**

Anlotinib hydrochloride placebo used in the control group in this study is tested by Chia Tai Tianqing Pharmaceutical Group Co., Ltd. to ensure the identification of the appearance, packaging, and smell. Random numbers are generated by the statistician of Nanjing Medical University using SAS software. The preparation of drug blinding codes and a blind letter is completed by personnel unrelated to this clinical trial. After the drug packaging, the blinding code will be stored in duplicate at Sun Yat-sen University Cancer Center and the sponsoring unit respectively.

The investigators, subjects, sponsor, and those responsible for the trial are blinded to the treatments of each participant. It is very important and must be done to maintain the blind status of the whole trial.

- 1. **Handling of** **randomization errors**

If a random error in subjects that do not meet the inclusion/exclusion criteria occurred, or incorrect treatment is scheduled, or enrolled subjects do not meet inclusion/exclusion criteria, the sponsor's representative and the investigator must discuss whether to continue or withdraw from the study. Sponsors are required to ensure appropriate record-keeping of this decision.

1. Subjects
   1. **Number of subjects**

The subjects are treat-naive patients with stage IIIB-IV EGFR-mutated NSCLC and it is expected that 310 subjects will be enrolled.

- 1. **Inclusion criteria**

Subjects can participate in the study only if all the following criteria are met:

1. Age 18-75 years;
2. Eastern Cooperative Oncology Group-Performance status (ECOG-PS) of 0-1;
3. Predicted life expectancy of ≥12 weeks;
4. Pathologically confirmed diagnosis of stage IIIB (not suitable for radiotherapy)/IV NSCLC; at least one measurable lesion based on RECIST v1.1 (tumor lesions with the longest diameter ≥10 mm on CT scan, and lymph nodes with a short diameter ≥15 mm on CT scan; no local treatment such as radiotherapy and freezing for measurable lesions);
5. Patients with primary NSCLC harboring activating EGFR mutations (exon 19 deletion or 21 L858R point mutation) according to any validated method;
6. No previous chemotherapy or other targeted therapy;

Note: Treatments during neoadjuvant therapy are not considered in treatment regimens; patients relapsing within six months of the completion of neoadjuvant therapy (partially defined as first-line treatment) could not be enrolled in this study; if recurrence occurs after more than 6 months, neoadjuvant therapy is not included in the treatment regimens.

1. Patients who have previously received radiotherapy are eligible, with radiation therapy to <25% of bone marrow (Cristy and Eckerman 1987) and without full pelvic or chest irradiation; prior radiation therapy must have been completed at least 4 weeks before enrollment and acute toxicity induced by radiation therapy must have recovered; local lesions that were treated with radiation could not be included in the measurable lesions unless significant progression of the lesion was recorded after the last radiotherapy;
2. Adequate function of the important organs as evidenced by the following:

- **Hemanalysis (no blood transfusion within 14 days, no correction with G-CSF or other hematopoietic stimulation factors):**

1. absolute neutrophil count (ANC) ≥1.5×10^9^/L;
2. platelets (PLT) ≥100×10^9^/L;
3. hemoglobin (Hb) ≥100g/L;

- **Biochemistry:**

1. total bilirubin (TBIL) <1.5×ULN；
2. alanine aminotransferase (ALT) and aspartate aminotransferase (AST) <2.5×ULN; ALT and AST <5×ULN in patients with liver metastases;
3. blood Urea Nitrogen (BUN) and creatinine (Cr) ≤1×ULN or creatinine clearance rate ≥50ml/min (Cockcroft-Gault formula);
4. Women of reproductive age must have already used effective contraception or have negative serum or urine pregnancy test results within 7 days before enrollment and be willing to use an adequate method of contraception during the trial period and 8 weeks after the last drug administration. For men, consent is given to use an appropriate method of contraception or to have been surgically sterilized during the trial period and 8 weeks after the last drug administration;
5. Be willing and able to provide written informed consent for the trial, and comply with all aspects of the protocol.
   1. **Exclusion criteria**

Subjects who meet any of the following criteria are not eligible to enter the study:

1. Small cell lung cancer (including mixed small cell and NSCLC);
2. Symptomatic brain metastases (patients with stable brain metastases who have completed treatment 21 days before enrollment are eligible, but have no symptoms of cerebral hemorrhage confirmed by brain MRI, CT, or venography);
3. Tumor lesion of ≤5 mm from the large blood vessels, or central tumor invading the local large blood vessels, or significant pulmonary cavitary or necrotic tumors by imaging (computed tomography [CT]/ magnetic resonance imaging [MRI]);
4. Patients with hypertension who are being treated with a combination of two or more antihypertensive drugs;
5. Patients with positive T790M mutations from genetic test;
6. Cardiovascular diseases: class II or more of myocardial ischemia or myocardial infarction, arrhythmia due to poorly controlled (including men with corrected QT interval [QTc] ≥450 ms and women with QTc ≥470 ms); patients in New York Heart Association (NYHA) class III-IV cardiac dysfunction, or left ventricular ejection fraction (LVEF) <50% by echocardiography;
7. Patients with a history of interstitial lung disease or concurrent interstitial lung disease;
8. Abnormal coagulation function (INR >1.5 or prothrombin time [PT] >ULN+4 seconds or APTT >1.5 ULN) with a bleeding tendency, or are receiving thrombolytic or anticoagulant therapy;
9. Hemoptysis (2 teaspoons or more per day) before enrollment;
10. Clinically significant bleeding symptoms or a definite bleeding tendency within 3 months before enrollment, such as gastrointestinal bleeding, bleeding hemorrhoids, gastric hemorrhagic ulcers, baseline levels for fecal occult blood (++ or more), or vasculitis;
11. Arterial and venous thrombosis within 12 months prior to enrollment, such as cerebrovascular accidents (including temporary ischemic attacks, cerebral hemorrhage, and cerebral infarction), deep vein thrombosis, and pulmonary embolism;
12. Known inherited or acquired bleeding and thrombophilia (e.g., hemophiliacs, coagulation dysfunction, thrombocytopenia, hypersplenism);
13. Unhealed wound or fracture for a long time (pathologic fracture caused by a tumor is not considered);
14. Major surgical operation, severe traumatic injury, fracture, or ulceration within 4 weeks of enrollment;
15. Factors significantly affecting oral medication such as swallowing difficulty, chronic diarrhea, and intestinal obstruction;
16. An abdominal fistula, gastrointestinal perforation, or intraperitoneal abscess within 6 months before enrollment;
17. Urinary protein ≥++, and confirmed 24-hour urinary protein ≥1.0 g;
18. Serous effusion with clinical symptoms requiring symptomatic treatment (including hydrothorax, ascites, and hydropericardium);

Note: Asymptomatic patients with serous effusions could be enrolled; symptomatic patients with serous effusions who are treated with symptomatic treatment (no anticancer drugs used for serous effusion), and then could be enrolled as judged by the investigators;

1. Active infections require antimicrobial therapy (e.g., antibacterial drugs, antiviral drugs, excluding anti-hepatitis B therapy for chronic hepatitis B, and antifungal drug treatment);
2. Active hepatitis B (HBV DNA ≥2000IU/mL or 104 copies/mL) or hepatitis C (hepatitis C antibody-positive, and HCV RNA above the lower limit of assay);
3. A history of psychotropic substance abuse with the inability to quit, or dysphrenia;
4. Participation in other clinical trials of antitumor drugs within 4 weeks prior to the study;
5. Patients with previous or co-existing uncured malignancies (other than skin basal cell carcinoma, cured cervical carcinoma in situ, and superficial bladder cancer);
6. Patients who had received a potent CYP3A4 inhibitor within 7 days prior to randomization, or had received a potent CYP3A4 inducer within 12 days prior to the study;
7. Pregnant or lactating women; A fertile patient who is unwilling or unable to use effective contraception;
8. The investigator identifies other conditions that may affect the proceeding of the clinical study and its outcomes.
   1. **Dropout/removal criteria**
9. Patients who failed to meet the inclusion criteria;
10. Safety evaluation could not be performed without one dose of the drug;
11. Both modern Chinese medicine and immunomodulators approved by SFDA are simultaneously used for the treatment of lung cancer;
12. The dose, method, and course of treatment are not specified according to the study protocol.
    1. **Termination criterion**
13. Withdrawl of informed consent and request to withdraw from the study;
14. Radiographic evidence of disease progression;
15. Pregnancy events occurred during the study;
16. Subjects who were still unable to tolerate toxicity after dose adjustment;
17. Decisions to withdraw from study by the sponsor.
18. Study Drugs
    1. **Overview of drugs**
       1. **Drug information**

Study Drugs are produced by Chia Tai Tianqing Pharmaceutical Group Co., Ltd., and the detailed information is shown in Table 6.1.1.

**Table 6.1.1 Drug Information**

| **Drugs** | **Specification** | **Drug storage** | **Manufacturer** |
| --- | --- | --- | --- |
| Anlotinib | 12 mg/tablet  10 mg/tablet  8 mg/tablet | Store in a sealed and dark place below 25°C | Chia Tai Tianqing Pharmaceutical Group Co., Ltd. |
| Placebo | 0 mg/tablet | Store in a sealed and dark place below 25°C | Chia Tai Tianqing Pharmaceutical Group Co., Ltd. |
| Gefitinib | 250 mg/tablet | Store at a room temperature of 15-30°C | Chia Tai Tianqing Pharmaceutical Group Co., Ltd.  Astrazeneca Pharmaceuticals Co., Ltd. |

Anlotinib and placebo are produced by Chia Tai Tianqing Pharmaceutical Group Co., Ltd., and batch numbers and expiration dates are detailed in the drug test report. Gefitinib tablets are produced by Chia Tai Tianqing Pharmaceutical Group Co., Ltd. or purchased from Astrazeneca Pharmaceuticals Co., Ltd.

- - 1. **Drug packaging**

Anlotinib is packaged in capsules, with 7 tablets per plate and 2 plates per small box.

- 1. **Dose and administration regimens**

Group A (experimental group): 250 mg gefitinib, po, qd + 12 mg anlotinib hydrochloride capsule, po, qd, D1-D14; administered under fasting conditions (taking medication at the same time every day), every 3 weeks;

Group B (control group): 250 mg gefitinib, po, qd + anlotinib hydrochloride mock capsule, 1 tablet, po, qd, D1-D14; administered under fasting conditions (taking medication at the same time every day), every 3 weeks.

Anlotinib is given at a dose of 12 mg as the initial dose (po, qd, D1-D14) every 21 days and the drug combination is used until disease progression, intolerable toxicity, withdrawal of consent, or drug discontinuation according to the investigator's judgment. If the administration is suspended due to no recovery of drug toxicity, treatment cycle is recalculated according to the regimen of consecutive 2 weeks and stop 1 week, while the follow-up period is still calculated according to the screening date. If patients discontinue anlotinib due to intolerable toxicity, gefitinib can be continued until disease progression.

**Note:** In principle, drug administration should be terminated for progressive disease (PD) patients; however, given the therapeutic characteristics of molecularly targeted drugs, the evaluation of cytotoxic chemotherapy drugs has certain defects. After the use of molecular targeted therapy in some patients, although the tumor increases, the internal tissues of the tumor may show obvious necrosis or degeneration; CT can show a decrease density within the tumor, which is generally believed that patients may benefit from the treatment. According to this protocol, for patients with obvious necrosis or degeneration of the tumor tissue and continuous improvement or stability of clinical tumor-related symptoms, if the patient voluntarily continues administration and there may be a survival benefit according to the principal investigator’s judgment, treatment can be continued until intolerable toxicity or disease progression. Close observation is required during administration.

- 1. **Drug administration, distribution, and recovery**

The study drug is governed by special personnel, and the investigator must ensure that all drugs are used only for the subjects enrolled in the study and that the dose and use are in accordance with the protocol. The remaining drugs shall be returned to Chia Tai Tianqing Pharmaceutical Group Co., Ltd., and study drugs shall not be transferred to any non-clinical participants.

The drug receipt shall be signed by two persons in duplicate, with each party (the clinical research unit and Chia Tai Tianqing Pharmaceutical Group Co., Ltd.) holding one copy. At the end of the study, the remaining drugs and empty boxes are collected, and the drug recovery sheet is signed by both parties. The distribution and collection of each drug should be recorded in a special record sheet timely.

The inspector is responsible for supervising the supply, use, storage of study drugs, and disposal of the remaining drugs.

- 1. **Destruction of study drugs**

The total quantity of study drugs shall be 120% of the design quantity, and the remaining drugs shall be returned to t Chia Tai Tianqing Pharmaceutical Group Co., Ltd. after the end of the study, which shall be recovered by the inspectors.

The investigator may destroy the used study drugs (non-state controlled chemicals) and empty boxes/bottles after written notification to the co-organizer without endangering the health of the population. The investigator should keep all study drug-handling records.

These records must document the evidence and quantity of each batch of destroyed drugs, disposal methods (as required by local law), and the person who handled the study drugs.

1. Dose Adjustment and Discontinuation
   1. **Dose adjustment of anlotinib**

The initial dose of anlotinib or placebo is 12 mg/day, and the anlotinib dose can be reduced to 10 mg/day for the first dose and 8 mg/day for the second dose. For patients unable to tolerate a dose of 8 mg/day, anlotinib should be discontinued and gefitinib should be administered alone until PD or intolerable toxicity.

|  | **Study group (anlotinib)** | **Control group (placebo)** |
| --- | --- | --- |
| Initial dose | ■■ 12 mg/day, qd | □□ 12 mg/day, qd |
| First dose reduction | ● 10 mg/day, qd | ○ 10 mg/day, qd |
| Second dose reduction | ■ 8 mg/day, qd | □ 8mg/day, qd |
| Third dose reduction | Discontinuation | Discontinuation |

- 1. **Dosage suspension and reduction**

If dosage suspension caused by drug toxicity has not recovered, the duration of dosage suspension should not exceed 6 weeks per cycle. Follow-up visits will be conducted at least every 1-2 weeks during discontinuation and the drug will be restarted at the original dose or a lower dose level after recovery of toxicity.

The subjects cannot return to the previous dosing pattern after dosage reduction and a maximum of three dose reductions are allowed in anlotinib therapy.

When patients experienced grade 3 hematological toxicities and grade 2 non-hematological toxicities, dosage suspension and reduction are considered; Dosage suspension will be performed in non-hematological toxicities including controllable nausea, vomiting, and fever with a definite cause (below 38°C) until the toxicity became tolerable. Aggressive symptomatic management without dosage suspension and reduction can also be conducted.

- - 1. **Standardization of dose suspension and reduction**

To ensure consistency of dosage adjustment, dosage suspension is performed at the time of dosage adjustments for each cycle. The criteria of dose suspension and reduction are as follows (once the criteria of dosage reduction are met which is considered poorly tolerated, a reduction should be performed).

**Dose Adjustment Criteria**

| **Toxicities graded by NCI** | **Hematological toxicities** |
| --- | --- |
| Grade I | Maintain the original dose (appropriate symptomatic treatment should be provided). |
| Grade II | Maintain the original dose (appropriate symptomatic treatment should be provided). |
| Grade III | Initially, suspend the drug and aggressive symptomatic treatment should be performed, and maintaining the original dose if toxicity restores to grade <II. |
| Grade IV | Initially, suspend drug and aggressive symptomatic treatment should be performed, and downward dose adjustment should be performed if toxicity restores to grade <II. |
|  | **Non-hematological toxicities** |
| Grade I | Maintain the original dose (appropriate symptomatic treatment should be provided). |
| Grade II | Symptomatic treatment and continue drug treatment should be provided. If AE resolves to grade <I within 2 weeks, continue drug treatment; If AE does not improve or aggravate, drug interruption until AE resolves to grade <I and continue drug treatment with original dose level. |
| Grade III | Drug interruption and symptomatic treatment should be provided. If AE resolves to grade <I within 2 weeks, continue drug treatment with the original dose level or at a dose reduced by one level by the investigator's discretion; Drug interruption is recommended if a grade 3 or above adverse reaction occurs. |
| Grade IV | Drug interruption and symptomatic treatment should be provided, and the dose is reduced by one level if AE resolves to grade <I; Drug interruption is recommended if a grade V AE occurs (such as Grade V kidney injury, neurotoxicity, cardiotoxicity, hepatotoxicity, and other life-threatening). |
| **AEs of special interest** | |
| Once hypertensive intracerebral hemorrhage, intracerebral hemorrhage, pulmonary hemorrhage (≥grade II), other hemorrhages (≥ grade III), arterial thrombosis, venous thrombosis (grade IV), Leukoencephalopathy syndrome, and gastrointestinal perforation occur, drug interruption is recommended and aggressive symptomatic treatment should be provided. | |

- 1. **Dose adjustment of gefitinib**

If the patient exhibits intolerable diarrhea (generally associated with dehydration), skin toxic effects, or other AEs caused by gefitinib, briefly discontinuing (up to 42 days) of gefitinib may be performed and then resuming the dose to 250 mg/day for the patient.

In the event of pulmonary symptoms (dyspnea, cough, and fever) gefitinib therapy should be interrupted, and a prompt investigation of these symptoms should occur. If interstitial lung disease is confirmed, gefitinib should be discontinued and appropriate treatment should be conducted.

No dosage adjustment is recommended for patients with different body weights, gender, ethnicity, renal function, or moderate to severe hepatic impairment due to liver metastases.

For patients treated with gefitinib combined with anlotinib who discontinue gefitinib therapy, the decision of proceeding with anlotinib is determined by the investigator.

When AEs occur, dose adjustment of gefitinib and subsequent dosing regimens are determined by the investigator.

- 1. **Management of anlotinib-related AEs**

Management of anlotinib-related AEs will be performed by the investigator based on the actual clinical situation (reference the following management).

1. **Fatigue**

Fatigue is often associated with oncologic treatment, and it is recommended to monitor the onset of severe fatigue by observation and documentation of the patient's fatigue. No dosage adjustment is necessary if grade 1-2 fatigue occurs; aggressive symptomatic treatment should be provided if grade 3-4 fatigue occurs. In absence of jaundice, thrombi, and pregnancy, progesterone drugs (such as megestrol) and multivitamins may be administered to help reduce fatigue and enhance physical performance. In addition, care must always be taken to identify other causes of fatigue such as those secondary to hypothyroidism, depression, anemia, or pain.

1. **Diarrhea**

Diarrhea may impact the digestive function, fluid and electrolyte balance of the patient, and the absorption of medications, early treatment should be performed. Diarrhea usually appears during the first few days of treatment. No dosage adjustment of anlotinib should be performed.

No dose adjustment of anlotinib should be required if grade 1-2 diarrhea occurs, and patients are recommended to: regulate dietary habits, drink more water, and eat easily digestible, absorbed, and vitamin-rich food; avoid intake of diarrhea-inducing diets (e.g., greasy, spicy foods, and coffee) or undergo medications such as stool softeners and gastrointestinal prokinetic agents; increase the diet with plenty of dietary fiber and administer micro-ecological drugs (e.g., bifid triple viable). Among them, when grade 2 diarrhea occurs, loperamide, diphenoxylate, gastric mucosal protective agents (montmorillonite powder), and berberine can be appropriately administered. Aggressive symptomatic treatment should be performed if grade 3-4 diarrhea occurs, and care must always be taken to replenish water and electrolytes to maintain fluid and electrolyte balance and provide acid-base disturbance. Immediately discontinue anlotinib until diarrhea is significantly reduced or ceases, and reduce the dose appropriate when restoring drugs.

1. **Hypertension**

Hypertension is emerging as one of the most common AEs. The [cardiovascular toxicities](https://www.sciencedirect.com/topics/medicine-and-dentistry/cardiovascular-toxicity) panel of the NCI recommends that patients with hypertension at baseline should be diagnosed before VEGF/VEGFR-inhibitor therapy, and patients should have a sustained blood pressure of <140/90 mmHg during the treatment. Blood pressure (BP) should be performed monitored before administration of the drug and especially in the first 2 weeks of treatment (daily monitoring should be performed), and monitoring must continue throughout treatment. Therefore, for patients with hypertension, the antihypertensive drugs should be administered based on individualized principles before receiving anlotinib to achieve early and favorable BP control (BP of ≤140/90 mmHg). However, prophylactic antihypertensive therapy is not recommended for patients with normal BP. Patients should be adequately informed to contact the investigator immediately for guidance when BP is >140/90 mmHg or when symptoms associated with elevated BP occur (e.g., significant headache, dizziness, visual disturbances, etc.). It has been suggested that antihypertensive drugs should be chosen according to the 2010 Chinese guidelines for the management of hypertension, regarding the risk factors for cardiovascular disease in patients. For patients combined with proteinuria, an angiotensin-converting enzyme inhibitor (ACEI) or an angiotensin receptor blocker (ARB) is recommended. A word of caution in drug selection is required that anlotinib is primarily metabolized by the lung CYP3A4 enzyme. Non-dihydropyridine calcium channel blockers (verapamil and diltiazem) can moderate CYP3A4 inhibition and are not recommended for BP control.

If a grade 3-4 increase in BP occurs, antihypertensive treatment or dose modification of this product should be performed under the guidance of the specialist. Drug discontinuation is recommended if the adverse reaction persists. This product should be permanently discontinued in patients who develop a hypertensive crisis. Recommendations for the prevention and treatment of anlotinib-associated hypertension can be viewed in **Table 7.4.1** hypertension classification criteria (NCI-CTC AE 4.0) and recommendations for prevention and treatment.

When hypertension occurs in [vital signs measurement](https://www.mdpi.com/748232), patients should rest for 15 minutes before BP is measured again. Hypertension has been identified as an AE if hypertension recurs and reaches grade II.

AEs are documented as one AE when the time interval between two hypertensive episodes is less than 1 week, and the highest grade of AE will be recorded with the time of onset defined as the time of the first hypertensive episode and the time of end defined as the time of outcome.

**Table 7.4.1. Classification criteria (NCI-CTC AE 4.0) and recommendations for prevention and treatment**

| Criteria | Definition | Prevention and treatment |
| --- | --- | --- |
| Grade I | SBP 120-139 mmHg, or DBP 80-89 mmHg | Close monitor of BP; Limit sodium intake, and quit smoke and alcohol; Continue to administer anlotinib without dose adjustment. |
| Grade II | SBP 140-159 mmHg, or DBP 90-99 mmHg | Close monitor of BP; Continue to administer anlotinib without dose adjustment; Treatment with antihypertensive drugs, and should not be stopped arbitrarily. |
| Grade III | SBP ≥160 mmHg, or DBP ≥100 mmHg | Suspension of anlotinib; Consider the combination of drugs for hypertension with poor control of monotherapy; Consult a cardiovascular specialist; Close monitor of BP; Continue the drug treatment at a dose reduced by one level with well-controlled blood event |
| Grade IV | Life-threatening (malignant hypertension or  Hypertensive emergencies/persistent neurological deficit) | Immediate and permanent drug discontinuation; Consult a cardiovascular specialist; Aggressive treatment and close monitor of BP and other vital signs |
| Grade V | Death | - |

1. **HFSR**

Patients should avoid mechanical trauma to hands and feet in the treatment, wearing loose-fitting comfortable shoes, softy cotton socks or gloves, and gel inserts, and vigorous exercise which may help limit the reaction; Avoiding direct sunlight on the skin, and using topical emollients or other preparations containing urea or lanolin to protect shin; Avoiding eating hot and spicy food. Treatment of moderate HFSR (if occurs) includes strengthening skin care by mild moisturizer or lubricant to keep the skin clean and moist and avoid secondary infection, pressure, or friction; Topical administration of exfoliating drugs may be administered (such as creams and ointments containing urea, 5% salicylic acid, and corticosteroid lotions), and dissolving the magnesium sulfate in warm water and soaking the affected skin if necessary; Topical antifungals or antibiotics; Appropriately administrating of B vitamins (B1, B6, and riboflavin) and celecoxib. If hyperkeratosis leads to thickened and calloused of foot skin, podiatrists can perform podiatric care to prevent further aggravation and use moisturizing ointment immediately after the pedicure.

The incidence of HFSR decreased with increasing treatment duration in the treatment period. Discontinuing the drug if grade >2 AEs appears three times and tends to worsen, and continue drug treatment if AE resolves to normal or grade 1; If HFSR has no further occurrence, the dose of anlotinib should progressively increase until the standard dose; If grade >2 AEs reoccurs after the resumption of dosing, it is recommended to continue dosing after a dose reducing by one level; If grade 3 of AEs persists or tend to worsen, permanent discontinuation of anlotinib is recommended. Recommendations for the prevention and treatment of anlotinib-associated HFSR can be viewed in **Table 7.4.2** HFSR classification criteria (NCI-CTC AE 4.0) and recommendations for prevention and treatment.

**Table 7.4.2. HFSR classification criteria (NCI-CTC AE 4.0) and recommendations for prevention and treatment**

| Criteria | Definition | Prevention and treatment |
| --- | --- | --- |
| Grade 1 | Minimal skin changes or dermatitis (e.g., localized erythema, hyperkeratosis, oedema, and painless), but not interfering with daily life | Continuing anlotinib administration without dose adjustment; Treating with topical medication at the first sign of symptoms. |
| Grade 2 | Significant skin changes (flaking, blisters, swelling, and hyperkeratosis) and pain, interfering with daily life and activities. | Continuing anlotinib administration with appropriate dose adjustment; Local drug; Oral B vitamins and celecoxib, and may combine with anti-inflammatory or anti-infective drugs. |
| Grade 3 | Severe skin changes (flaking, blisters, bleeding, ulcers, oedema, and hyperkeratosis) with significant pain and limited personal self-care ability. | Suspension of anlotinib; Analgesic management and topical treatment; Combination of anti-inflammatory or anti-infective drugs; Administering a lower dose of anlotinib if symptoms resolve; Discontinuing anlotinib if symptoms persist and worsen. |

1. **Proteinuria**

When grade >2 proteinuria occurs, the dose of anlotinib is interrupted and awaiting for recovering. If grade 2 or higher proteinuria occurs, continue drug treatment of anlotinib after a dose reducing by one level; if proteinuria persists and worsens, discontinue the drug.

In the event of renal insufficiency or nephrotic syndrome, the drug must be discontinued immediately and aggressive symptomatic treatment must be administered. There is no definitive treatment for proteinuria induced by anti-angiogenic inhibitors, but ACEI and ARB analogues may be administered as appropriate due to their ability in reducing intrarenal tubular pressure and thus proteinuria, as well as reduce possible cardiac AEs. Recommendations for the prevention and treatment of anlotinib-associated proteinuria can be viewed in Table 7.4.2 proteinuria classification criteria (NCI-CTC AE 4.0) and recommendations for prevention and treatment.

**Table 7.4.3. Proteinuria classification criteria (NCI-CTC AE 4.0) and recommendations for prevention and treatment**

| Criteria | Definition | Prevention and treatment |
| --- | --- | --- |
| Grade 1 | Urokinase protein (+) or 24-hour urine protein excretion <1.0 g | Continuing anlotinib administration without dose adjustment |
| Grade 2 | Urokinase protein (++) or 24-hour urine protein excretion of 1.0-3.4 g | Continuing anlotinib administration without dose adjustment; Drug intervention should be considered; Monitor 24-hour urine routine and 24-hour urine protein excretion |
| Grade 3 | 24-hour urine protein excretion >3.4 g | Suspension of anlotinib; Consultation with a nephrologist specialist; Drug intervention should be performed; Dose reduction of anlotinib after recovery of proteinuria to ≤ grade 2; If grade 3 proteinuria still occurs after 2 dose reductions, anlotinib therapy should be permanently discontinued |

1. **Bleeding**

The PT and international normalized ratio (INR) should be closely monitored during the administration of anlotinib to monitor patients for bleeding tendencies and related symptoms; the drug should be interrupted if a grade 3-4 bleeding event occurs. If [upper gastrointestinal bleeding](https://www.sciencedirect.com/science/article/pii/S0025712508000023) occurs, anlotinib must be interrupted and aggressively treated according to clinical protocols should be performed.

1. **Thrombus**

If any arterial thrombosis (e.g., cerebral ischemia, stroke, angina, myocardial infarction) occurs, drug administration should be discontinued immediately and the study should be withdrawn. If symptomatic grade IV venous thrombosis occurs, drug administration should be discontinued in patients, with withdrawal from the study.

For patients with thrombotic symptoms, symptomatic treatment, surgery, or anticoagulant drugs should be scheduled immediately.

For patients with venous thrombosis, the criteria for dose adjustment are as follows:

| **Venous thrombosis** | **Dose adjustment** |
| --- | --- |
| Grade II | Anlotinib is maintained at the original dose level and closely monitored. |
| Grade III or asymptomatic grade IV | 1. Anlotinib is discontinued. 2. Symptomatic treatment with anticoagulants (small-molecular-weight heparin) 3. Anticoagulants can be administrated for at least one week and no severe (grade III, IV) bleeding patients are found after symptomatic improvement of thrombosis, with continued administration after a reduced dose level according to the investigator’s judgment. |

1. **Reversible posterior leukoencephalopathy syndrome**

Reversible posterior leukoencephalopathy syndrome (RPLS) has not been reported in the completed clinical trials of anlotinib, but has been reported in the clinical use of marketed anti-neovascularization antibodies and small molecule inhibitors.

The clinical manifestations of RPLS are headache, altered/unclear consciousness, abnormal vision/blindness, and convulsions, which are often accompanied by hypertension. At present, the etiology of RPLS is unknown. Once suspected symptoms occur, the administration should be discontinued immediately with symptomatic treatment and strict control of blood pressure. Once confirmed by imaging, patients are withdrawn from the clinical trial.

1. **Abdominal pain**

Abdominal pain is uncommon in the treatment of lung cancer with anlotinib, and most patients have tumor-associated symptoms. Meanwhile, gastrointestinal perforation has been reported occasionally in clinical trials of anlotinib and other types of antiangiogenic drugs. For patients with abdominal pain, investigators should be aware of potential gastrointestinal perforation. Once gastrointestinal perforation occurs, the administration should be discontinued immediately, withdrawal from the trial, with symptomatic treatment.

1. **Pulmonary interstitial fibrosis**

The clinician should fully understand the patient's condition and be familiar with the drugs that may induce pulmonary toxicity. The clinical symptoms and chest X-ray or CT changes are carefully observed. Once patients have unexplained symptoms such as cough, chest tightness, suffocation, dyspnea, and hemoptysis, the causes should be identified in time, and administration should be discontinued as soon as possible after excluding other causes (such as infection and heart failure). Bronchoalveolar lavage and surgical lung biopsy are important methods for the diagnosis of interstitial lung disease. At present, there is no satisfactory treatment for pulmonary fibrosis. It is recommended to correct hypoxemia and timely use glucocorticoids referring to the guidelines for the diagnosis and treatment of idiopathic pulmonary (interstitial) fibrosis issued by the Respiratory Society of Chinese Medical Association.

**Note:** If necessary, specialist consulting supports the diagnosis and treatment.

1. Concomitant Medications
   1. **Drugs prohibited or used with caution during the study**
      1. **Medications that interfere with hepatic cytochrome P450 enzymes**

CYP3A inducers (dexamethasone, carbamazepine, rifampin, and phenobarbital), inhibitors (ketoconazole, itraconazole, erythromycin, and clarithromycin), CYP3A4 substrate (simvastatin, cyclosporine, and piperidine), other medications metabolized by CYP3A4 (benzodiazepines, dihydropyridine calcium antagonist, calcium antagonists, and HMG-CoA reductase inhibitors), CYP2C9 substrate, and CYP2C19 substrate will be used cautiously.

| P450 enzyme | Substrate |
| --- | --- |
| CYP2C9 | Diclofenac, phenytoin, diclofenac, tolbutamide, and s-warfarin |
| CYP2C19 | Diazepam, promethazine, lansoprazole, and s-mephenytoin |

- - 1. **Medications that prolong the cardiac QTc**

Medications that prolong the cardiac QT will be used cautiously due to the toxicity of anlotinib will prolong the QT interval in clinical practice, and medications including but not limited to:

- Antimicrobials (clarithromycin, azithromycin, erythromycin, roxithromycin, metronidazole, and moxifloxacin);
- Antiarrhythmic drugs (quinidine, sotalol, amiodarone, disopyramide, and procaine amide);
- Antipsychotics (risperidone, fluphenazine, haloperidol, haloperidol, thioridazine, pimozide, olanzapine, and clozapine);
- Antifungal drugs (fluconazole and ketoconazole);
- Antimalarial drugs (mefloquine and chloroquine);
- Antidepressants (amitriptyline, imipramine, clomipramine, dosulepin, and doxepin).
  - 1. **Chinese medicine preparations and immunomodulators with anti-cancer effects**

This protocol prohibits the use the Chinese medicine preparations for the treatment of lung cancer that is approved by CFDA and immunomodulators (such as thymosin, interferon, interleukin-2 zilongjinpian, and fungal polysaccharides).

- 1. **Permitted concomitant medications/treatments**

All treatments that are considered by the investigator to have no effects on study endpoints may be administered, such as unconventional therapies (e.g., herbal or acupuncture) and vitamin/mineral supplements. Bisphosphonates are allowed for subjects with bone metastases during the trial. Palliative radiation therapy in a small area (<5% bone marrow region) will be permitted for subjects with uncontrolled pain of bone metastases after systemic therapy or topical analgesia. Antiviral medication should be administered during enrollment for patients with positive hepatitis B surface antigens during the screening period. Clinical co-morbidities and the emergence of various types of AEs should be treated aggressively. Any combination medications should be recorded in the electronic case report forms (eCRFs) in strict accordance with GCP regulations [[16](#_ENREF_16)].

1. Study Procedures

All patients must read and sign an informed consent form approved by the ethics committee before the initiation of the study. All study procedures are required to be performed according to the time window procedure in the schematic flow diagram (Appendix 2, flow diagrams of anlotinib in combination with gefitinib for NSCLC). Clinical observation and examination time is independent of the duration of drugs, and related examinations (including imaging examinations) are performed at each course of treatment.

- 1. **Screening**

The following procedures must be completed within 4 weeks before study drug initiation (unless specifically indicated).

(1) Sign the informed consent form;

(2) Demographic: Age, sex, occupation, nationality, height, and weight;

(3) Smoking history: Smoking duration, number of cigarettes (light smokers ≤15 cigarettes/day; medium smokers <25 cigarettes/day; heavy smokers ≥25 cigarettes/day)

(4) Tumor diagnosis: Date of diagnosis for clinically staged, histologic classification (large cell carcinomas and adenocarcinomas), pathological regional (primary and metastatic lesions), TNM classification, and clinical staging.

(5) Gene mutation status (the test results of mutation status must be provided if the patient has been tested).

- EGFR gene mutation status;
- Other gene mutation status (if have).

(6) History of antitumor therapy:

- Tumor surgery: The date and type of previous surgery;
- History of chemotherapy/targeted/immunotherapy (including neoadjuvant and adjuvant therapy): Drug names, dose, administration cycle, onset and duration, best efficacy, and reasons for changing drugs (if due to disease progression, imaging should be recorded; if due to intolerance, AEs and grade of AEs should be recorded).
- Radiotherapy history: radiotherapy time and radiotherapy dose.

(7) The date of the tumor progression or recurrence after the last treatment.

(8) Comorbidities: Such as diabetes, hypertension, or chronic obstructive pulmonary disease.

(9) BP monitoring: BP is measured by the investigator during the screening period; Coffee and tobacco intake are prohibited within 30 minutes before each BP is measured and the measurement is taken in the sitting position with the arm at heart level after at least 10 minutes of quiet sitting, and each BP measurement is taken on the same side.

(10) Imaging examination (CT or MRI): Enhanced CT of the chest, abdomen and enhanced MRI of cranial. For subjects with bone metastases, a whole-body bone scan should be performed; Patients with stable brain metastases should be confirmed without intracranial hemorrhage within 21 days before randomization (written informed consent must be obtained before performing any imagine examination in the trial, except that CT/MRI scan obtained before signing informed consent may be used for screening evaluation if they meet the criteria. The examination must be performed within the first 28 days of the date of the randomization.).

(11) Echocardiography: especially analysis of the values of LVEF (assessments before signing informed consent or within 28 days before randomization if necessary).

(12) Epidemiology: HBs-Ag, Anti-HCV, syphilis, and HIV. HBV DNA test is required if HBs-Ag positive, and HCV RNA test for anti-HCV antibody-positive patients.

(13) Concomitant medications and therapy: assessments of concomitant medication and concomitant therapy within 28 days before enrollment and during the trial period.

(14) Documentation of AEs: Record AEs from the first dose of the study drug.

The following screening procedures must be completed within 7 days before initiating drug therapy (Blood tests including hemanalysis, blood biochemistry, and coagulation function within 2 weeks before randomization is performed.):

(1) ECOG PS;

(2) Vital signs: Heart rate, respiratory rate, temperature, and BP;

(3) BP monitoring;

(4) Physical examination: Facial features, integumentary system, lymph nodes, eyes, ears, nose, throat, oral, respiratory system, cardiovascular system, abdomen, urinary tract system, musculoskeletal system, nervous system, and spiritual state;

(5) Hemanalysis: Hemoglobin, red blood cell, white blood cells, absolute neutrophil count, lymphocyte count, and platelet count;

(6) Urinalysis: Urine protein, glucose, occult blood (red blood cell and white blood cell), pH, and ketone bodies; If the semi-quantitative method shows the levels of protein ≥2+ (e.g., urine test strip), 24-hour urine analysis are performed and the 24-hour urine analysis of enrolled patients must be < 1g;

(7) Stool tests: occult blood;

(8) Blood biochemistry: TBil, ALT, AST, ALP, r-GT, total protein, Cr, UA, blood glucose, triglyceride, cholesterol, potassium, sodium, chlorine, calcium, urea, and phosphonium;

(9) Coagulation tests: PT, APTT, TT, Fbg, and INR;

(10) ECG: 12-lead ECG. A prolongation of PR-, QTc-, and QT-intervals should be observed and ECG can be performed 3 times consecutively to identify abnormalities (approximately 5-minute interval for each examination and a prolongation of QTc- should be indicated);

(11) Myocardial enzyme profile test: No more myocardial enzyme profile tests except for one test performed within 7 days before enrollment (testing is required if the ECG is abnormal).

(12) Pregnancy test (women of childbearing age);

(13) QoL: The questionnaire should be completed by the subject himself/herself. However, if the subject is unable to read or write, an authorized staff member or guardian could administer it.

The following screening procedures must be performed in screened patients.

1. Subjects who meet eligibility criteria will be randomized by a web-based system and assigned a subject number and treatment groups as well as obtain the dose of the study drug.
2. Study drug administration.
   1. **Treatment period**

Subjects who meet the criteria are administered the treatment in cycles of 21 days. The visits are conducted on days 21±3 of each cycle for the first 4 cycles, on days 42±3 per 2 cycles for the following 22 cycles, and on days 63±7 per 3 cycles from 27 cycles until the disease progresses or intolerable toxicity.

1. Vital signs: Heart rate, respiratory rate, temperature, and blood pressure;
2. Physical examination: Facial features, integumentary system, lymph nodes, eyes, ears, nose, throat, oral, respiratory system, cardiovascular system, abdomen, urinary tract system, musculoskeletal system, nervous system, and spiritual state;
3. BP monitoring: BP is measured by the subject himself/herself and recorded in the patient diary card. BP is tested at least 3 times per week for the first 2 cycles and followed up each day if the blood pressure was abnormal. In addition, BP is measured again by the investigator at each follow-up visit, coffee and tobacco intake are prohibited within 30 minutes before each BP is measured and the measurement is taken in the sitting position with the arm at heart level after at least 10 minutes of quiet sitting, and each BP measurement is taken on the same side;
4. Hemanalysis: Hemoglobin, red blood cell, white blood cells, absolute neutrophil count, lymphocyte count, and platelet count;
5. Urinalysis: Urine protein, glucose, occult blood (red blood cell and white blood cell), pH, and ketone bodies; If the semi-quantitative method shows the levels of protein ≥2+ (e.g., urine test strip), 24-hour urine analysis is performed;
6. Blood biochemistry: TBil, ALT, AST, ALP, r-GT, total protein, Cr, uric acid (UA), blood glucose, triglyceride, cholesterol, potassium, sodium, chlorine, calcium, urea, and phosphonium;
7. 12-lead ECG. A prolongation of PR-, QTc-, and QT-intervals should be observed. If symptoms such as precordial pain and palpitations occur, an ECG should be checked immediately, together with an additional cardiac enzyme profile; LVEF is required for ECG abnormalities with significant clinical significance.
8. Stool tests: Occult blood;
9. Coagulation tests: PT, APTT, TT, Fbg, and INR;
10. Imaging examination (cycle 1 days 21±7 and days 21±7 per 3 cycles from 27 cycles):

- The window duration of the image is not impacted by drug interruption and is performed according to the baseline;
- The target lesion identified at baseline should be examined under the same conditions as the baseline examination (layer thickness of the scan, use of contrast agent, etc.); Patients with bone metastases detected at baseline should be examined again and other lesions other than the above or new lesions suspected should also be detected.
- Unscheduled imaging may be performed when disease progression is suspected (e.g., worsening of symptoms) or when confirmation of PR or CR is required (6 weeks after achieved PR or CR);

1. QoL: Conducting by the investigator asking questions and the subject answering;
2. ECOG PS;
3. AEs: Recording AEs from the date of the first dose of the study drug to at least 30 days after the last dose and follow up until the AEs resolved or stabilized;
4. Concomitant medications: Concomitant medications and therapy during the trial should be recorded and if the subject discontinues treatment, the concomitant medication and therapy are recorded only in the case of a novel or unresolved AEs associated with treatment;
5. Study drug: Distribution and recovery of study drug.
   1. **End-of-treatment and withdrawal study**

At the end-of-treatment and withdrawal study, if the patient is not examined within 14 days before the end of treatment, the following tests should be performed.

1. ECOG PS;
2. Vital signs: Heart rate, respiratory rate, temperature, and BP;
3. Physical examination: Facial features, integumentary system, lymph nodes, eyes, ears, nose, throat, oral, respiratory system, cardiovascular system, abdomen, urinary tract system, musculoskeletal system, nervous system, and spiritual state;
4. BP monitoring: BP is measured by the investigator, coffee and tobacco intake are prohibited within 30 minutes before each BP is measured and the measurement is taken in the sitting position with the arm at heart level after at least 10 minutes of quiet sitting, and each BP measurement is taken on the same side;
5. Hemanalysis: Hemoglobin, red blood cell, white blood cells, absolute neutrophil count, lymphocyte count, and platelet count;
6. Urinalysis: Urine protein, glucose, occult blood (red blood cell and white blood cell), pH, and ketone bodies; If the semi-quantitative method shows the levels of protein ≥ 2+ (e.g., urine test strip), 24-hour urine analysis is performed;
7. Blood biochemistry: TBil, ALT, AST, ALP, r-GT, total protein, albumin, Cr, uric acid (UA), blood glucose, triglyceride, cholesterol, potassium, sodium, chlorine, calcium, urea, and phosphonium;
8. Stool tests: Occult blood;
9. Coagulation tests: PT, APTT, TT, Fbg, and INR;
10. 12-lead ECG;
11. Pregnancy test;
12. QoL;
13. AEs;
14. Recovery drug;
15. Imaging examination: If imaging is not performed within 4 weeks before the end of treatment, an imaging examination is required at the end of study treatment or withdrawal study. For patients with non-imaging progression (intolerable, other conditions), tumor evaluation performs every 2 cycles until disease progression, death, or the initiation of other oncologic therapy.
    1. **30-day post-withdrawal follow-up**

Safety assessment and follow-up of AEs should be continued for all subjects 30 days after the end of the last dose, with documentation of concomitant therapy.

1. Documentation of concomitant medications and therapy;
2. Follow-up of AEs.
   1. **Follow-up of survival**

Subjects who end the 30-day visit after drug discontinuation will start the follow-up of survival. Information of survival (record the date and cause of death) and end-of-treatment (including treatment) period is collected by telephone (asking the subjects and their relatives or a local physician) at least once every 3 months until death or subjects loss of visit or termination of the study by the sponsor. Each follow-up of survival is recorded in the follow-up format.

- 1. **Unplanned follow-up**

Subjects may experience AE requiring unplanned follow-up during the trial as follows.

1. Documentation of concomitant medications and therapy;
2. Follow-up of AEs
3. Documentation of relevant examinations (if have).
4. Study Assessments
   1. **Efficacy assessment**
      1. **Primary endpoint**

**PFS**

PFS is defined as the time from randomization to the first time of disease progression or death from any cause, whichever occurs first. If the subject did not experience disease progression or death, then PFS was defined as the date of the last confirmed progression-free. Patients who discontinued for reasons other than disease progression (no subsequent tumor image) and starting the therapy after the trial were censored at the time of discontinuation or initiation of treatment. In cases other than those described above, pre-planned sensitivity analyses will undertake to define PFS based only on the date of confirming tumor progression events. New onset other tumor is not considered indicative of PD and is not censored as data.

If the image examination and evaluation show progression, the data of disease progression is not the time showing signs of disease progression, but the time of the development of radiologically noticeable PD.

- - 1. **The secondary endpoints**

**OS**

OS is defined as the time from the randomization data to the date of death due to any cause. OS is censored at the date of the last visit for those still alive at the time of the last visit. Patients lost to follow-up were censored at the time of the last contact. Censored OS is defined as the time from randomization to censor.

**ORR**

The proportion of patients with confirmed tumor volume reduction to pre-specified values and maintenance of minimum time requirements, including the number of subjects with an objective response (PR or CR). Objective responses are assessed per RECIST v1.1. Participants must have at least one measurable lesion at baseline, and the efficacy criteria were classified as CR, PR, SD, and PD according to RECIST v1.1.

**DCR**

The percentage of subjects with the disease control (PR, CR, or SD [≥ 8 weeks]) will be presented.

**DOR**

The time from first documented CR or PR to disease progression or death from any cause, whichever occurred first (taking as reference for PD the smallest measurements recorded in the trial).

**TTPD**

TTPD is defined as the time from the date of enrollment to the first date of documented progression according to RECIST v1.1.

**QoL**

The changes in relevant clinical symptoms and objective examination results of patients before and after treatment were observed for scoring, and the scoring results of each domain of the scale were recorded in the eCRF according to the requirements of the quality of life scores. QoL is assessed by the EORTC QLQ-C30 (version 3) and EQ-5D.

- 1. **Safety analysis**
     1. **AEs**
        1. **Definitions of AEs**

AE is defined as any untoward medical occurrence in a patient or clinical investigation subject administered a pharmaceutical product and which does not necessarily have to have a causal relationship with this treatment. Any AEs that occur within 30 days from the initiation of the study drug to the end of treatment in this trial, regardless of whether or not have a causal relationship with this treatment, will be determined as AEs.

Investigators are required to record any AEs in subjects, including the events, AEs-related symptoms, time of occurrence, severity, duration, management, and outcomes.

- - - 1. **AEs grade**

The severity grade of any AEs will be assessed according to the definitions in NCI-CTCAE v4.0. For AEs that are not listed in the table, the following criteria can be referenced.

Grade I is defined as mild; asymptomatic or mild symptoms; clinical or diagnostic observations only; intervention not indicated.

Grade II is defined as moderate; minimal, local, or noninvasive intervention indicated; limiting age-appropriate instrumental activities of daily living (ADL, instrumental ADL refers to preparing meals, shopping for groceries or clothes, using the telephone, managing money, etc).

Grade III is defined as severe or medically significant but not immediately life-threatening; hospitalization or prolongation of hospitalization indicated; disabling; limiting self-care ADL (self-care ADL refers to bathing, dressing and undressing, feeding self, using the toilet, taking medications, and not bedridden).

Grade IV is defined as life-threatening consequences; urgent intervention is indicated.

Grade V is defined as death related to AE.

**Note:** For abnormal AEs or abnormal laboratory test indicators allowed by the inclusion criteria at the time of enrollment, the severity of the AEs and drug adjustments were performed according to the investigator’s discretion.

- - - 1. **Causality to study drugs**

An AE is any unintended medical occurrence in a clinical study after the signing of informed consent, which does not necessarily have a causal relationship with the treatment, regardless of whether it is assigned to the experimental groups or even the administration of drugs. Any abnormal changes in objective laboratory tests should be recorded faithfully, together with the time of occurrence, severity, duration, management, and outcomes. Relationships between the study drug and AEs were rated by the investigator as definitely, probably, possibly, unlikely, and unrelated according to five criteria. Definitely, probably, and possibly are classified as drug-related AEs and the incidence of AEs is calculated using the above three as the numerator and the total number of subjects in the safety assessment set as the denominator. The criteria of causality assessment are as follows.

**Table 11.3.1.3. Causality assessment of AEs and study drugs**

| Criteria | Definition |
| --- | --- |
| Definitely | The administration time and onset of the event exhibit a reasonable relationship, the suspected AEs fulfill the criteria for the typical reactions of the drug, the suspected AEs disappear or are mitigated after drug discontinuation, and the same AEs recurred after repetitive administration of the study drugs. |
| Probably | The administration time and onset of the event exhibit a reasonable relationship, a clinical course not consistent with the known effects, and the clinical status of the patient and other modalities may also produce the event. |
| Possibly | The event does not follow a sequence of time from drug administration, is not a previously known or suspected effect of the test drug, and the clinical status of the patient and other modalities may also produce the event. |
| Unlikely | The event does not follow a sequence of time from drug administration, is not a previously known or suspected effect of the test drug, the clinical status of the patient and other modalities may produce the event, the suspected AEs disappear or are mitigated after drug discontinuation, and the same AEs recurred after repetitive administration of the study drugs. |
| Unrelated | No clear relationship between the onset of the event and the administration time, a clinical course is consistent with the known effects, and other concomitant drug uses that may also produce the event |

- - 1. **SAEs**
       1. **Definitions of SAEs**

SAE is defined as any adverse event that results in any one of the following unanticipated events during the clinical trial period:

(1) Life-threatening events (immediate risk of death);

(2) Resulting in hospitalization or prolongation of existing hospitalization;

(3) Causing persistent or severe disability or incapacity;

(4) Causing congenital anomalies/birth defects;

(5) Drug overdose.

- - - 1. **Pregnancy**

Pregnancy in patients will be reported as SAEs.

- - - 1. **Disease progression**

Disease progression (including signs and symptoms of progression) under the trial will not be reported as SAEs, but death due to disease progression during the trial or safety reporting period will be reported as SAEs. Hospitalization due to signs and symptoms of disease progression will also not be reported as SAEs. During the trial or safety reporting period, events leading to death must be reported as SAEs.

- - - 1. **Undergo other anti-tumor treatments**

If the subject initiates other anti-tumor therapy, the AEs do not result in death are reported until the initiation of a new antineoplastic therapy. If death occurs within the entire SAE reporting period after the end of the study treatment, death must be reported regardless of whether the patient received additional treatment.

- - - 1. **Hospitalization**

AEs in clinical settings resulting in hospitalization or prolongation of existing hospitalization should be considered SAEs. Any initial admission (even if less than 24 hours) to a healthcare facility meets this criterion. Hospitalization excludes the following.

1. Rehabilitation facility;
2. Nursing facility;
3. Routine emergency room admissions;
4. Same-day surgeries (as outpatient/same-day/ambulatory procedures).

If hospitalization or prolongation of hospitalization does not result from worsening AEs, the AEs will not be reported as SAEs. Including:

1. Admission for treatment of a preexisting condition not associated with the development of a new AE or with a worsening of the preexisting condition (e.g., for work-up of persistent pre-treatment lab abnormality);
2. Administrative admission (e.g., for the yearly physical exam);
3. Protocol-specified admission during a clinical trial (e.g., for a procedure required by the trial protocol);
4. Optional admission not associated with a precipitating clinical adverse event (e.g., for elective cosmetic surgery);
5. Pre-planned treatments or surgical procedures should be noted in the baseline documentation for the entire protocol and/or for the individual subject;
6. Admission exclusively for the administration of blood products.

Diagnostic and therapeutic non-invasive and invasive procedures, such as surgery, should not be reported as AEs. However, the medical condition for which the procedure was performed should be reported and it meets the definition of AEs. For example, acute appendicitis that begins during the AEs reporting period should be reported as AEs, and the resulting appendectomy should be recorded as treatment of the AEs.

- - - 1. **SAEs reporting requirements**

SAEs should be reported from the date the subject signs the informed consent until 30 (inclusive) calendar days after the last dose of the study drug. If an SAE occurs, the clinical monitor and principal investigator are to be notified within 24 hours of awareness of the event. Meanwhile, fill out, sign and date the SAE form, and immediately report it by fax to the sponsor, the team leader, the ethics committee, the SFDA, and the FDA of the (province or city) of the researcher's region.

SAEs should be reported to the sponsor within 24 hours during a period of continued administration of drugs after the end of the study. Information on all SAEs is required to be recorded in the SAE form. SAEs occurring within 30 days of the last dose must be reported and occurring after 30 days of the last dose are generally not reported unless suspected to be related to the study drug.

SAEs should be recorded faithfully, together with the time of occurrence, severity, duration, management, and outcomes. If the investigator considers that an SAE is unrelated to the study drug but is potentially related to the clinical process (e.g., discontinuation of treatment, or comorbidity during the trial), this relationship should be detailed in the narrative section of the SAEs page of the CRF. If any changes are made to the grade of SAEs and the causality to study drugs during the study, changes must be reported to the sponsor. All SAEs should be followed up until recovery or stabilization of the disease.

- - - 1. **SAEs reporting procedures**

Any SAE occurring during the clinical period and within 30 days after discontinuation of the drug must be reported immediately to the CRA of the sponsor, the PI of the clinical study unit, and the ethics committee, as well as, report to the State Food and Drug Administration Drug Registration Division, safety supervision department, and Health administrative department, respectively, within 24 hours. Meanwhile, the investigator must complete an SAE Form with a detailed description of the time of SAE occurred, its severity, its causality to study drugs, and the interventions performed, and sign the report.

**Table 11.3.2.7. SAE contact**

| Unit | Contact | Telephone/Fax |
| --- | --- | --- |
| Sun Yat-sen University Cancer Center | Ethics Committee | 020-87343535 |
| Chia Tai Tianqing Pharmaceutical Group Co., Ltd. | Medical Affairs | 025-68551504 |
| State Food and Drug Administration Drug  Registration Division | | 010-68313344-1003/010-88363228 |
| Division of Medical Care, Department of  Medical Administration, Ministry of Health | | 010-68792413 |

- - 1. **Process for unblinding**

Investigators are not permitted to unblind if there is a necessary medical reason arises. Unblinding will only be granted by the study center director and principal investigator in case of an emergency such as SAE. The investigator should inform the CRA, the medical director of the sponsor, and the ethics committee to log into the BioKnow-RTS system if blindness is broken. The date, subjects, reason, and process for unblinding must be recorded in the EDC and simultaneously reported to the SAE.

- 1. **Drug resistance mechanisms**

This study will perform a multigene test (10 mL of peripheral blood each time before treatment, at the first efficacy evaluation, and at the time of tumor progression) on the peripheral blood of patients by QiYuan 329 gene panel (OrigiMed, Shanghai, China) for monitoring the resistance mechanism of targeted therapy

1. Ethical Considerations and Informed Consent
   1. **Ethical considerations**

The protocol is approved by the ethical committee of Sun Yat-sen University Cancer Center. The sponsor and the investigator should provide documents such as Application Form for Ethical Review, List of Clinical Trial Study Team Members, Certificate of Analysis, Sample of Informed Consent, Phase I Clinical Trial Protocol, CRF, and Investigator's Brochure. This trial will be conducted in accordance with the ethical principle of the Declaration of Helsinki (version 1996), GCP issued by CFDA, and related regulations. The investigator will not begin any study subject activities until approval from the Clinical Research Ethics Committee of the Clinical Trial Team leader. Any modifications of the protocol during the clinical study should be declared and filed to the ethics committee.

- 1. **Informed consent**

Subjects must provide informed consent to participate in this trial before receiving treatment in this trial to guarantee their legal rights. It is the responsibility of the investigator to provide an adequate explanation in understandable language of the aims, importance, anticipated benefits, potential hazards, and consequences of the study. If the subjects and their legally acceptable representative (LAR) are unable to read/write, then an impartial witness should be present during the entire informed consent process and must append his/her signatures to the consent form on that day. Informed consent documents must be marked version and date or modification date.

1. Sponsor/Investigator Responsibilities

To ensure the quality processes, the sponsor and investigator should strict adherence to GCP.

- 1. **Sponsor**

(1) Clinical research associates (CRA) will provide the investigator with the investigator’s brochure or drug instructions and explain the protocol and eCRF completion to the investigator before clinical initiation. The clinical supervisor is responsible for clinical monitoring and regular visits, with the following visit frequencies: At least one visit per cycle for the first 4 cycles; At least one visit every 2 cycles after 5 cycles.

(2) CRA should ensure that they can keep in touch with the investigator at any time by telephone, fax, and mail.

- 1. **Investigator**

(1) Investigator must responsible for obtaining an informed consent form signed by each subject or their representative.

(2) Investigators must seriously follow the requirements to fill out eCRF.

(3) Investigator must actively cooperate with CRA for regular visits.

(4) Investigator must ensure the completeness of laboratory records, clinical records, and original medical records.

1. Data Management
   1. **Data collection**

In this protocol, the eCRF is used for clinical research data capture, and study staff member whose training in this function are authorized to log in to the electronic data capture (EDC) system. The PI or designated clinical research coordinator (CRC) should enter the data into the EDC system in accordance with the follow-up requirement and eCRF completion guide logical verification procedure implemented in the system typically runs data check to assure the completeness and logicality of the study-related records in EDC system and notice the eCRF entry error (if any), and PI or the CRC can make appropriate corrections and explanation. The PI will get CD-ROM or certified copy containing the patient data after the database is locked for record-keeping.

The investigator is ultimately responsible for data collection and report of all clinical and laboratory data recorded in the eCRF and other data collection forms (original records), to assure the attributable, legible, contemporaneous, original, accurate, enduring, complete, and consistent.

The eCRF is required to be signed by the investigator or the investigator’s designated representative and data recorded in eCRF needs to be verified to ensure credibility. Any change or correction to an eCRF and source documents should be dated, signed, and explained (if necessary), and should not obscure the original entry.

As a rule, the original record is the diagram of the hospital or doctor, and data collected by eCRF should be consistent with them. In some instances, however, the capture of study data as the original record can also be performed by eCRF and should be archived by the research institution for accurate documentation.

- 1. **Statistical analysis**
     1. **Analysis datasets**

1. Full analysis set (FAS)

FAS included all randomly allocated patients who were treated with at least one dose of the study drug and received at least one post-treatment efficacy assessment, according to the intention to treat (ITT) principle. No imputation of missing data.

1. Per-protocol set (PPS)

PPS will be defined as all subjects who met the trial protocol, did not take prohibited drugs, with favorable compliance, and completed CRF. No imputation of missing data. Efficacy analysis was conducted based on PPS populations.

1. Safety analysis set (SS)

The SS population will serve as the population for the safety analysis. All randomized subjects have received at least one dose of the research drug and have a post-administration safety record.

- - 1. **Statistical analysis plans**

All statistical analysis will be performed using the SAS statistical package (≥9.3 version) with a two-sided test. P ≤0.05 is considered significant, with 95% confidence intervals (CI).

1. Baseline

Quantitative data including age, height, weight, and so on are described as mean ± standard deviation (SD), median, maximum, and minimum values, and qualitative data such as gender and ECOG score are reported by number and frequency.

1. Efficacy assessment

The primary endpoint of this trial, PFS, will be estimated using the Kaplan-Meier method with a 95% CI and presented with the Kaplan-Meier curve. The comparison of PFS between intervention arms will be performed using a stratified log-rank test. The HR and the corresponding 95% CI will also be provided based on a Cox proportional hazard model, using such as age, ECOG PS, and EGFR mutations (exon 19 deletions or an exon 21 mutation) as stratification factors.

The secondary endpoint such as OS, TTPD, and DoR will be analyzed in the same method as the primary endpoint. The cochran-mantel-haenszel (CMH) method or Chi-square test will be used to compare ORR and DCR between the groups, and Fisher's exact test method will be used to calculate ORR, DCR, and 95% CI. The Student t-test or Wilcoxon rank sum test will be used to compare the QoL between the groups.

1. Safety analysis

Safety analysis will be mainly descriptively summarized and the mean or incidence of pre- and post-administration examination tests (if necessary). Vital signs, physical examination, and laboratory tests will be demonstrated mainly through the changes from baseline in clinical and the incidence of abnormalities. All AEs will be coded using the Medical Dictionary for Regulatory Affairs (MedDRA) for safety analysis. Summary and compare the incidence of all AEs, treatment-emergent adverse events (TEAEs) with grade 3 or more, serious TEAEs, and so on between two intervention arms will be tabulated according to MedDRA PT and primary system organ class (SOC), as well as CTCAE grading.

- 1. **Interim analysis**

The interim analysis will be conducted by IDMC and will conduct 1-2 analyses. The IDMC will analyze the safety and efficacy data (PFS) and advises on the study conducted by the results of interim analyses. The IDMC, consisting of 3-4 independent oncologists and one independent statistician, is independent of the sponsors and investigators.

- 1. **Data storage**

All documents must be archived by the investigator. According to the requirements of GCP, data will be stored under lock and key for 5 years.

- 1. **Publication policy**

All data will be published only with the consent of the PI.

References

[1] Landis SH, Murray T, Bolden S, Wingo PA, Cancer statistics, 1999, CA: A cancer Journal for Clinicians. 49 (1999) 8-31.

[2] Novello S, Le Chevalier T, Chemotherapy for non-small-cell lung cancer. Part 1: Early-stage disease, Oncology (Williston Park, NY). 17 (2003) 357-64.

[3] Schiller JH, Harrington D, Belani CP, Langer C, Sandler A, Krook J, et al., Comparison of four chemotherapy regimens for advanced non-small-cell lung cancer, The New England journal of medicine. 346 (2002) 92-8.

[4] Fukuoka M, Wu YL, Thongprasert S, Sunpaweravong P, Leong SS, Sriuranpong V, et al., Biomarker analyses and final overall survival results from a phase III, randomized, open-label, first-line study of gefitinib versus carboplatin/paclitaxel in clinically selected patients with advanced non-small-cell lung cancer in Asia (IPASS), Journal of clinical oncology : official journal of the American Society of Clinical Oncology. 29 (2011) 2866-74.

[5] Mitsudomi T, Morita S, Yatabe Y, Negoro S, Okamoto I, Tsurutani J, et al., Gefitinib versus cisplatin plus docetaxel in patients with non-small-cell lung cancer harbouring mutations of the epidermal growth factor receptor (WJTOG3405): an open label, randomised phase 3 trial, The Lancet Oncology. 11 (2010) 121-8.

[6] Maemondo M, Inoue A, Kobayashi K, Sugawara S, Oizumi S, Isobe H, et al., Gefitinib or chemotherapy for non-small-cell lung cancer with mutated EGFR, The New England journal of medicine. 362 (2010) 2380-8.

[7] Han JY, Park K, Kim SW, Lee DH, Kim HY, Kim HT, et al., First-SIGNAL: first-line single-agent iressa versus gemcitabine and cisplatin trial in never-smokers with adenocarcinoma of the lung, Journal of clinical oncology : official journal of the American Society of Clinical Oncology. 30 (2012) 1122-8.

[8] Ettinger DS, Wood DE, Akerley W, Bazhenova LA, Borghaei H, Camidge DR, et al., Non–small cell lung cancer, version 6.2015, Journal of the National Comprehensive Cancer Network. 13 (2015) 515-24.

[9] Petrelli F, Borgonovo K, Cabiddu M, Barni S, Efficacy of EGFR tyrosine kinase inhibitors in patients with EGFR-mutated non-small-cell lung cancer: a meta-analysis of 13 randomized trials, Clinical lung cancer. 13 (2012) 107-14.

[10] Mok TS, Wu YL, Thongprasert S, Yang CH, Chu DT, Saijo N, et al., Gefitinib or carboplatin-paclitaxel in pulmonary adenocarcinoma, The New England journal of medicine. 361 (2009) 947-57.

[11] Sandler A, Gray R, Perry MC, Brahmer J, Schiller JH, Dowlati A, et al., Paclitaxel-carboplatin alone or with bevacizumab for non-small-cell lung cancer, The New England journal of medicine. 355 (2006) 2542-50.

[12] Zhou C, Wu YL, Chen G, Liu X, Zhu Y, Lu S, et al., BEYOND: A Randomized, Double-Blind, Placebo-Controlled, Multicenter, Phase III Study of First-Line Carboplatin/Paclitaxel Plus Bevacizumab or Placebo in Chinese Patients With Advanced or Recurrent Nonsquamous Non-Small-Cell Lung Cancer, Journal of clinical oncology : official journal of the American Society of Clinical Oncology. 33 (2015) 2197-204.

[13] Seto T, Kato T, Nishio M, Goto K, Atagi S, Hosomi Y, et al., Erlotinib alone or with bevacizumab as first-line therapy in patients with advanced non-squamous non-small-cell lung cancer harbouring EGFR mutations (JO25567): an open-label, randomised, multicentre, phase 2 study, The Lancet Oncology. 15 (2014) 1236-44.

[14] Li J, Qin S, Xu J, Xiong J, Wu C, Bai Y, et al., Randomized, Double-Blind, Placebo-Controlled Phase III Trial of Apatinib in Patients With Chemotherapy-Refractory Advanced or Metastatic Adenocarcinoma of the Stomach or Gastroesophageal Junction, Journal of clinical oncology : official journal of the American Society of Clinical Oncology. 34 (2016) 1448-54.

[15] Syed YY, Anlotinib: First Global Approval, Drugs. 78 (2018) 1057-62.
